# Aza-Henry Reactions on *C*-Alkyl Substituted Aldimines

**DOI:** 10.3390/molecules21060723

**Published:** 2016-06-02

**Authors:** Alessia Pelagalli, Lucio Pellacani, Elia Scandozza, Stefania Fioravanti

**Affiliations:** Dipartimento di Chimica, Università degli Studi di Roma “La Sapienza”, P.le Aldo Moro 5, I-00185 Roma, Italy; alessia.pelagalli@uniroma1.it (A.P.); lucio.pellacani@uniroma1.it (L.P.); eliasca93@gmail.com (E.S.)

**Keywords:** nitro compounds, amines, carbon–carbon bond formation

## Abstract

The reactivity of *C*-CH_3_ substituted *N*-protected aldimines in aza-Henry addition reactions was compared with that of the analogous trifluoromethylated compounds. *C*-Alkyl aldimines easily reacted with nitro alkanes under solvent-free conditions and in the absence of catalyst, despite being worse electrophiles than *C*-CF_3_ aldimines, they gave the aza-Henry addition only when ZrCl_4_ was added. The presence of a bulky group on the imine carbon deeply influenced the reactivity.

## 1. Introduction

The development of stereoselective reactions to create carbon–carbon bonds between compounds bearing a heteroatom functionality can provide valuable building blocks for organic synthesis. Starting from this purpose and considering the importance of the 1,2-diamine structural motif in biologically active natural products, drugs, and more recently as chiral auxiliaries and chiral ligands in asymmetric catalysis, general methods to synthesize this class of compounds are most relevant. Among them, the aza-Henry reaction [1,2,3,4], also called nitro-Mannich reaction, involves the nucleophilic addition of nitro alkanes to aldimines and leads to the synthesis of β-nitro amines, valuable compounds containing two vicinal nitrogenated functionalities with different oxidation states. Also for this, the aza-Henry reaction presents important synthetic applications [5,6,7,8], providing access to a wide variety of other organic compounds by functional transformations of the nitro group into other chemical functionalities, such as amines, carbonyl groups, hydroxylamines, oximes, and nitriles. 

While only recently, *C*-CF_3_ substituted aldimines [9,10,11] are reported in the aza-Henry reactions as interesting trifluoromethyl nitrogen-containing starting materials; the reactivity of *C*-aryl analogues has been well documented in the literature, while very few data are reported on the reactivity of *C*-alkyl substituted aldimines [2]. Classically, non-enantioselective nucleophilic addition of nitro alkanes to *C*-aryl substituted aldimines usually required the presence of a base as catalyst [12]. On the contrary, the same reaction on *C*-trifluoromethyl imines takes place only in the presence of a Lewis acid, namely ZrCl_4_, which was found to be the best catalyst for that reaction [9,10].

Stimulated by this difference of reactivity, we thought it might be interesting to deepen the study of the behavior of *C*-alkyl substituted aldimines in nitro-Mannich reactions, especially to compare their reactivity with that of fluorinated analogues. In fact, as is well known [13,14,15,16,17,18,19,20], the presence of fluorine atoms influences both the reactivity and biological properties of compounds in which they are present.

## 2. Results and Discussion

Different *C*-alkyl substituted *N*-protected aldimines **3a**–**i** were synthesized by solvent-free equimolar direct condensation reactions between primary amines **1a**–**c** and aldehydes **2a**–**c** (Table 1) following the methodology previously reported by us to obtain trifluomethylated substrates in high yields [21]. The reactions proceeded with high stereoselectivity, leading, in high yields and purity, only to (*E*)-aldimines, which can be used in the subsequent aza-Henry additions without further purification.

**Scheme 1:**
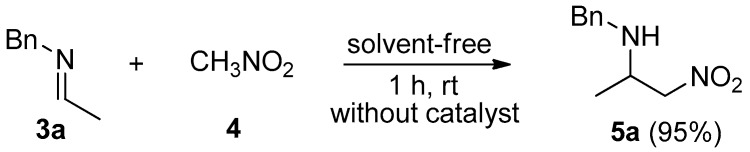
Best reaction conditions for the aza-Henry addition.

The nitro-Mannich addition of nitromethane (**4**) on aldimine **3a**, which were selected as suitable reactants to fix the best reaction conditions, was tested under solvent-free conditions either in the presence or in the absence of a catalyst (base or acid). The reactions carried out by using different organic (TEA, DABCO) or inorganic (K_2_CO_3_, KF) bases did not give any product of addition, leading only to a very complex reaction mixture with the disappearance of all reagent signals in the NMR spectra. On the contrary, performing the reaction with ZrCl_4_, while the nitromethane was quantitatively recovered, the ^1^H-NMR spectrum of the crude mixture showed only the disappearance of the imine signals, whose hydrolysis is mainly promoted by the presence of Lewis acid. Only working without added catalyst, the expected product **5a** was obtained after 1 h of stirring at room temperature by a self-catalyzed addition in which aldimine **3a** acts as both base and electrophile [22,23] (Scheme 1).

**Scheme 2:**
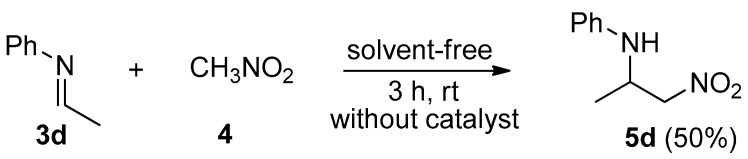
Aza-Henry addition on anil **3d**.

To the best of our knowledge, neither the *C*-aryl nor the *C*-trifluoromethyl substituted aldimines have been reported to react in the absence of catalyst, thus confirming the strong influence of the imido carbon substituent on the reactivity of aldimines, that may affect the reaction outcome by influencing the electrophilicity of the *sp*^2^ carbon and/or making the nitrogen lone pair more or less available. To partially confirm this, we decided to test the reactivity of anil **3d**. While similar *N*-aryl trifluoromethyl aldimines did not give nitro-Mannich addition either without catalyst or in the presence of ZrCl_4_ [9], *N*-phenyl *C*-methyl aldimine (**3d**) lead to the expected β-nitro amine **5d**, in the absence of catalyst, although in longer times and lower yields with respect to *N*-alkyl substituted **3a** (Scheme 2).

The last result seems to confirm the relevant role of the substituent nature on imine carbon. In fact, the EDG methyl (electron donating group, EDG), counteracting the mesomeric aromatic effect of the phenyl residue, permitted that the self-catalyzed addition reaction also takes place on an aldimine derived from a primary aromatic amine.

Then, by turning to match the diastereoselective reaction outcome, the chiral imine (*R*)-**3g** was reacted with nitro alkanes **4** and **6** to study both the *syn*/*anti* and the stereoselective facial outcome of the nitro-Mannich additions. The results were compared with those already reported for the same aza-Henry reactions performed on chiral trifluoromethyl aldimine **3j** [9] (Table 2).

**Figure 1:**
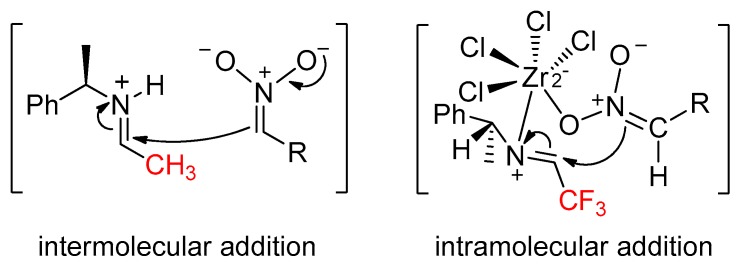
Different possible mechanisms.

The replacement of the −CF_3_ group with the −CH_3_ group on the imine carbon seems to partially influence time and yields of additions (Table 1, entries 1, 3), but much more the *syn*/*anti* reaction control, probably due to the different involved mechanism. In fact, while nitro alkanes added to imine **3g** through an intermolecular self-catalyzed reaction, imine **3j** undergoes addition only in the presence of ZrCl_4_ by an intramolecular process involving the *in situ* formation of a chiral zirconated intermediate (Figure 1).

**Figure 2:**
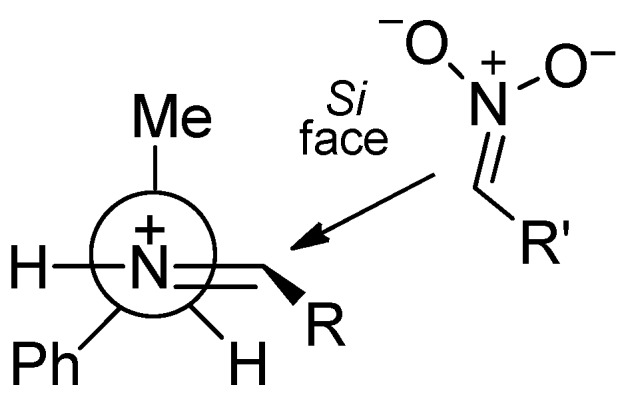
Stereoselective intermolecular attack.

As shown in Table 2, the chiral intermediate permits a partial control of the *syn*/*anti* diastereoselectivity that was completely lost by an intermolecular reaction approach. On the contrary, the aldimine chiral resident center leads to the same stereoselective facial attack, the β-nitro amines always being obtained with the same dr. By 2D NOESY analyses (see Supplementary Materials) [10] the *R* configuration to the new chiral center of major isomers **7′g** (entry 2), *syn***-8/8′g**, and *anti***-9/9′g** were assigned. The obtained data showed that the nucleophilic intermolecular attack takes place preferentially on the methyl aldimine *Si* face (Figure 2), just like the intramolecular addition proceeds preferentially on the analogous trifluoromethyl aldimine *Si* face.

**Scheme 3:**
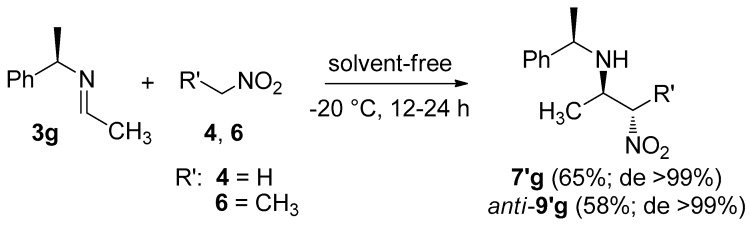
Temperature effect on the stereoselective aza-Henry additions.

Finally, the temperature effect was considered. As previously reported [10], the stereoselective outcome of addition reactions carried out on trifluoromethyl imines was not affected by the temperature. In fact, while working at low temperatures (from 0 to −20 °C) only a significant decrease in yields and no changes in diastereoselectivity was observed. Instead, a very high complete stereoselectivity control was registered when the nitro-Mannich reactions were performed on (*R*)-**3g** under low temperature and the chiral β-nitro amines **7′g** and *anti*-**9′g** were obtained as diastereomerically pure compounds, although in lower yields and longer reaction times (Scheme 3).

The stereochemical results can be due to different steric and electronic effects of two considered groups. In fact, the volume of the fluorine atom is close to that of the hydrogen atom and, above all, the –CF_3_ group is one of the strongest electron-withdrawing groups able to increase the carbon electrophilicity [24,25,26]. As a possible consequence, it seems to be more difficult to control the nucleophilic attack rate and even stereoselectivity starting from trifluoromethyl aldimine **3j**.

Continuing our study, we decided to consider the influence of two other different alkyl groups on the reactivity of *C*-alkyl substituted aldimines with nitro alkanes.

**Scheme 4:**
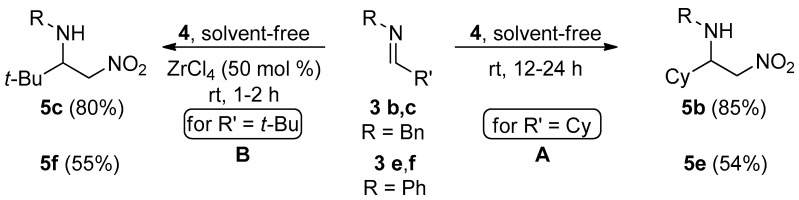
Reactivity of different *C*-alkyl substituted aldimines with CH_3_NO_2_ (**4**).

Therefore, imines **3b**,**e** derived from cyclohexanecarbaldehyde and imines **3c**,**f**, derived from pivalaldehyde (Table 1) were reacted under solvent-free conditions with nitromethane (**4**). Unexpectedly, while *C*-hexyl imines reacted without catalyst giving the corresponding β-nitro amines **5b**,**e** (Scheme 4, **A**), the presence of the *tert*-butyl group on the imine carbon required ZrCl_4_ as catalyst to lead to the expected compounds **5c**,**f** (Scheme 4, **B**), with no reaction taking place either without catalyst or in the presence of a base (K_2_CO_3_ or Et_3_N), or also by changing other reaction parameters (molar ratios, temperature (from 25 to 60 °C), presence of a solvent (THF, CH_2_Cl_2_)).

The different reactivity of aldimines **3c**,**f**, derived from pivalaldehyde appeared very intriguing. In fact, the *tert-*butyl group, characterized by an electron-donating effect but even by a strong steric hindrance, seems to influence the C=N reactivity in the same way of the −CF_3_ group, that on the contrary, as reported before, is a well-known strong EWG (electron withdrawing group, EWG) although with limited steric hindrance. Therefore, trifluoromethyl aldimines can be considered good electrophiles, unlike their unfluorinated analogues.

A possible explanation for this unexpected similar reactivity can be proposed.

As already reported by us [10] in the reaction of trifluoromethyl aldimine **3j** the catalyst is required to increase the acidity of the nitro alkane α-proton by ZrCl_4_-coordination, since the presence of the −CF_3_ group increases the C=N double bond electrophilicity, but at the same time drastically decreases the lone pair nitrogen availability so that the nucleophile may not be formed.

**Figure 3:**
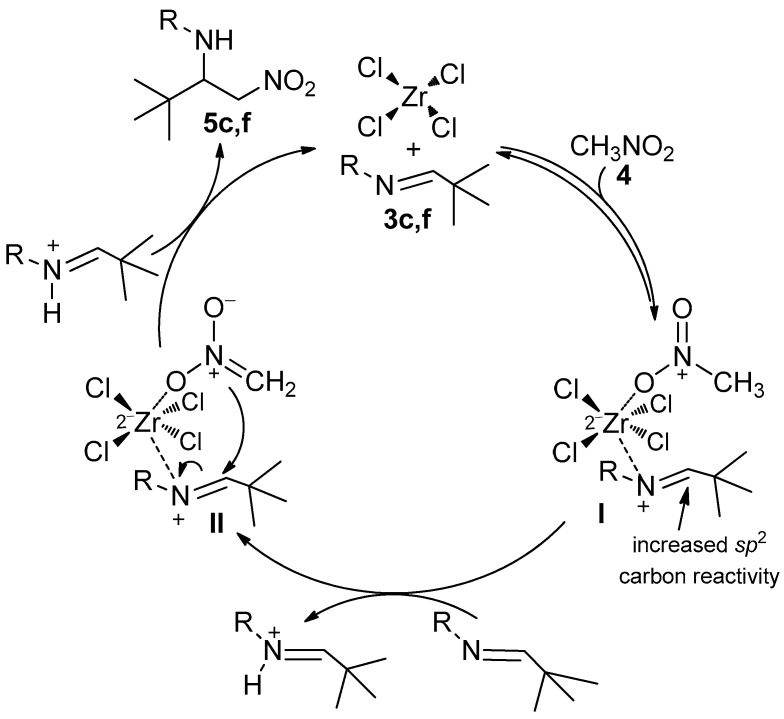
Proposed outcome for the ZrCl_4_-catalyzed addition.

On the contrary, the presence of a *tert*-butyl group in **3c**,**f** favors the nitronate formation but could decrease the reactivity of the C=N carbon due to both steric and electronic effects. Therefore, the ZrCl_4_-coordination with aldimine **3** and nitromethane **4** (**I**) increases the *sp*^2^ carbon reactivity but, above all, allows the reaction to be able to occur through an intramolecular addition (Figure 3), as already proposed to explain the reactivity of *C*-CF_3_ substrates, thereby probably minimizing the *tert*-butyl steric effect.

Finally, the stereoselective reaction outcome was even studied on *C*-alkyl substituted (*E*)-aldimines **3h**,**i**. The reactions were performed at low temperature and starting from **3i** in the presence of ZrCl_4_ as catalyst. The results are reported in Table 3.

The increase of steric hindrance on the imine carbon influenced the course of the aza-Henry additions. Besides determining an increase of reaction time and a decrease in the yields, the addition of **6** failed in the *syn*/*anti* stereocontrol on the aldimine **3h** (entry 2) and does not take place starting from the highly hindered *tert*-butyl aldimine **3i** (entry 3). Only the stereoselectivity of attack remains very high giving only one diastereomer by addition of nitromethane (**4**) (entries 1, 3). The same selectivity was observed in the reaction of **6** with aldimine **3g** (entry 3), with only one diastereomer of *syn*/*anti* geometric isomers being formed.

## 3. Experimental Section

IR spectra were recorded on a Perkin-Elmer 1600 FT/IR spectrophotometer in CHCl_3_ as solvent. ^1^H-NMR and ^13^C-NMR spectra were recorded on a VARIAN XL-300 spectrometer at 300 and 75 MHz or on a Bruker Avance III at 400 and 101 MHz, respectively, at room temperature. CDCl_3_ was used as solvent and CHCl_3_ and CDCl_3_ as internal standard for ^1^H and ^13^C, respectively. The NOESY experiments were performed with a Bruker Avance III spectrometer at 400 MHz using CDCl_3_ as solvent and CHCl_3_ as internal standard and used to assist in structure elucidation [27]. HPLC analyses were performed with a Varian 9001 instrument using an analytical column (3.9 × 300 mm, flow rate 1.3 mL/min; detector: 254 nm) equipped with a Varian RI-4 differential refractometer, or a Varian 9050 UV/VIS detector. Eluents were HPLC grade. HR-MS analyses were performed using a Micromass Q-TOF Micro quadrupole-time of flight (TOF) mass spectrometer equipped with an ESI source and a syringe pump. The experiments were conducted in the positive ion mode. Optical rotation was determined at 25 °C with a JASCO DIP-370 polarimetry at a wavelength of 589 nm, using a quartz cell of 1 cm length.

### 3.1. General Procedure for the Synthesis of C-alkyl Imines (***3a**–**i***)

Equimolar amounts (5 mmol) of aldehyde and amine were reacted under solvent free conditions. The reaction mixtures were stirred at room temperature for 15 min, then CH_2_Cl_2_ (3 mL) and anhydrous sodium sulfate (Na_2_SO_4_) were added and the mixtures were filtered off. The organic solvent was evaporated under vacuum to give the expected aldimines which were used without further purification. **3a**–**i** [28,29,30,31,32,33,34,35] are known compounds.

### 3.2. General Procedure for the Synthesis of C-alkyl β-nitro Amines

*Procedure A.* (*E*)-Aldimines **3** (1 mmol) were stirred at room temperature (1–18 h) with a five-fold excess of nitro compound **4** (eight-fold excess of nitro compound **6)** under solvent-free conditions. After removal of nitro compound excess under vacuum, the crude mixtures were purified by flash chromatography on silica gel.

*Procedure B.* As procedure A, but at −20 °C.

*Procedure C.* ZrCl_4_ (0.5 mmol) was added to a mixture of (*E)*-aldimine **3i** (1 mmol) and nitro compound **4** (5 mmol) or **6** (8 mmol). The reactions were performed under solvent-free conditions and stirred at room temperature (1–2 h). Then, after addition of water (5 mL), the crude mixtures were extracted three times with Et_2_O. The collected organic layers were dried over anhydrous Na_2_SO_4_ and the solvent evaporated under vacuum. The crude mixtures were purified by flash chromatography on silica gel.

*Procedure D.* Same as procedure C, but at −20 °C.

*N-Benzyl-1-nitropropan-2-amine (***5a**, *Procedure A).* Yellow oil; (0.165 g, 85%); purified by flash chromatography on silica gel (eluent: hexane/ethyl acetate = 80:20); IR ν_max_ 3355, 1571 cm^−1^; ^1^H-NMR (CDCl_3_, 300 MHz) δ 7.31–7.18 (5H, m), 4.37–4.26 (m, 2H), 3.83–3.67 (2H, m), 3.43–3.32 (1H, m), 1.59 (1H, br), 1.15 (3H, d, *J* = 6.6 Hz); ^13^C-NMR (CDCl_3_, 75 MHz) δ 139.6, 128.4 (2C), 127.9 (2C), 127.1, 80.2, 51.2, 50.8, 18.2; HRMS *m*/*z* [M + H]^+^ 195.1139 (calcd for C_10_H_15_N_2_O_2_, 195.1134). 

*N-Benzyl-1-cyclohexyl-2-nitroethanamine (***5b***, Procedure A)*. Yellow oil; (0.223 g, 85%); purified by flash chromatography on silica gel (eluent: hexane/ethyl acetate = 80:20); IR ν_max_ 3353, 1562 cm^−1^; ^1^H-NMR (CDCl_3_, 300 MHz) δ 7.31–7.18 (5H, m), 4.43 (1H, dd, *J* = 11.5, 5.0 Hz), 4.34 (1H, dd, *J* = 11.7, 7.8 Hz), 3.75 (2H, s, *J* = 10.3 Hz), 3.14–3.08 (1H, m), 1.78–1.45 (7H, m), 1.25–0.90 (5H, m); ^13^C-NMR (CDCl_3_, 75 MHz) δ 139.8, 128.3 (2C), 128.0 (2C), 127.0, 77.1, 60.8, 51.5, 39.7, 29.0, 28.8, 26.2, 26.1 (2C); HRMS *m*/*z* [M + H]^+^ 263.1758 (calcd for C_15_H_23_N_2_O_2_, 263.1760).

*N-Benzyl-3,3-dimethyl-1-nitrobutan-2-amine (***5c***, Procedure C)*. Pale yellow oil; (0.189 g, 80%); purified by flash chromatography on silica gel (eluent: hexane/ethyl acetate = 80:20); IR ν_max_ 3359, 1571 cm^−1^; ^1^H-NMR (CDCl_3_, 300 MHz) δ = 7.35–7.22 (5H, m), 4.59 (1H, dd, *J* = 11.8, 4.0 Hz), 4.32 (1H, dd, *J* = 11.8, 8.8 Hz), 3.87–3.77 (2H, m,), 3.16 (1H, dd, *J* = 8.8, 4.0 Hz), 1.75 (1H, br), 0.97 (9H, s); ^13^C-NMR (CDCl_3_, 75 MHz) δ 140.1, 128.4 (2C), 128.3 (2C), 127.2, 78.3, 65.5, 54.5, 35.4, 26.6 (3C); HRMS *m*/*z* [M + H]^+^ 237.1601 (calcd for C_13_H_21_N_2_O_2_, 237.1603). 

*N-(1-Nitropropan-2-yl)aniline (***5d***,* *Procedure A).* Yellow oil; (0.09 g, 50%); purified by flash chromatography on silica gel (eluent: hexane/ethyl acetate = 80:20); IR ν_max_ 3355, 1567 cm^−1^; ^1^H-NMR (CDCl_3_, 300 MHz) δ ^1^H NMR (CDCl_3_, 300 MHz) δ 7.22 (2H, t, *J* = 7.8 Hz) 6.79 (1H, t, *J* = 7.3 Hz), 6.67 (2H, d, *J* = 7.9 Hz), 4.58 (1H, dd, *J* = 12.2, 4.5 Hz), 4.38 (1H, dd, *J* = 11.7, 8.3 Hz), 4.26–4.20 (1H, m), 3.71 (1H, br), 1.38 (3H, d, *J* = 6.3 Hz); ^13^C-NMR (CDCl_3_, 75 MHz) δ 145.6, 129.6 (2C), 118.9, 113.7 (2C), 79.2, 47.8, 18.6; HRMS *m*/*z* [M + H]^+^ 181.0982 (calcd for C_9_H_13_N_2_O_2_, 181.0977).

*N-(1-Cyclohexyl-2-nitroethyl)aniline (**5e**, Procedure A).* Pale yellow oil; (0.134 g, 54%); purified by flash chromatography on silica gel (eluent: hexane/ethyl acetate = 80:20); IR ν_max_ 3356, 1564 cm^−1^; ^1^H NMR (CDCl_3_, 300 MHz) δ 7.19 (2H, t, *J* = 7.8 Hz), 6.75 (1H, t, *J* = 7.3 Hz), 6.66 (2H, d, J = 7.9 Hz), 4.63–4.39 (2H, m), 4.12–3.88 (1H, m), 3.72 (1H, br), 1.98–1.50 (6H, m), 1.38 –0.95 (5H, m); ^13^C NMR (CDCl_3_, 75 MHz) δ 146.5, 129.5 (2C), 118.4, 113.4 (2C), 76.4, 56.9, 40.6, 29.6, 28.8, 26.1, 25.9 (2C); HRMS *m*/*z* [M + H]^+^ 249.1601 (calcd for C_14_H_21_N_2_O_2_, 249.1603).

*N-(3,3-Dimethyl-1-nitrobutan-2-yl)aniline (***5f**, *Procedure C).* Pale yellow oil; (0.122 g, 55%); purified by flash chromatography on silica gel (eluent: hexane/ethyl acetate = 90:10); IR ν_max_ 3350, 1563 cm^−1^; ^1^H-NMR (CDCl_3_, 400 MHz) δ 7.16 (2H, t, *J* = 7.9 Hz), 6.72 (1H, t, *J* = 7.3 Hz), 6.67 (2H, d, *J* = 7.8 Hz), 4.63 (1H, dd, *J* = 12.1, 4.8 Hz), 4.36 (1H, dd, *J* = 12.1, 8.6 Hz), 4.10 (1H, m), 3.63 (1H, br), 1.02 (9H, s); ^13^C-NMR (CDCl_3_, 100 MHz) δ 147.4, 129.5 (2C), 118.4, 113.4 (2C), 77.2, 61.2, 29.8, 26.6 (3C); HRMS *m*/*z* [M + H]^+^ 223.1451 (calcd for C_12_H_19_N_2_O_2_, 223.1447).

*(S)-1-Nitro-N-[(R)-1-phenylethyl]propan-2-amine (**7g***, *Procedure A).* Yellow oil; (0.135 g, 65%); separated by HPLC (eluent: hexane/ethyl acetate = 90:10); [α]_D_: −55.4 (*c* = 40 g/100 mL, CHCl_3_); IR ν_max_ 3358, 1555 cm^−1^; ^1^H-NMR (CDCl_3_, 300 MHz) δ 7.37–7.27 (5H, m), 4.41 (1H, dd, *J* = 11.4, 5.4 Hz), 4.23 (1H, dd, *J* = 11.4, 5.4 Hz), 3.93 (1H, q, *J* = 6.5 Hz), 3.28–3.14 (1H, m), 1.79 (1H, br), 1.34 (3H, d, *J* = 6.5 Hz), 1.15 (3H, d, *J* = 6.6 Hz); ^13^C-NMR (CDCl_3_, 75 MHz) δ 144.7, 128.6 (2C), 127.2, 126.5 (2C), 81.2, 55.3, 49.2, 25.1, 17.6; HRMS *m*/*z* [M + H]^+^ 209.1297 (calcd for C_11_H_17_N_2_O_2_, 209.1290).

*(R)-1-Nitro-N-[(R)-1-phenylethyl]propan-2-amine (***7′g***, Procedure B). * Yellow oil; (0.168 g, 81%); purified by flash chromatography on silica gel (eluent: hexane/ethyl acetate = 90:10); [α]_D_: ‒70.4 (*c* = 40 g/100 mL, CHCl_3_); IR ν_max_ 3358, 1555 cm^−1^; ^1^H-NMR (CDCl_3_, 300 MHz) δ 7.37–7.22 (5H, m), 4.42 (1H, dd, *J* = 11.4, 5.4 Hz), 4.33 (1H, dd, *J* = 11.3, 5.3 Hz), 3.90 (1H, q, *J* = 6.5 Hz), 3.32–3.14 (1H, m), 1.58 (1H, br), 1.33 (3H, d, *J* = 6.5 Hz), 1.12 (3H, d, *J* = 6.6 Hz); ^13^C-NMR (CDCl_3_, 75 MHz) δ 145.1, 128.6 (2C), 127.2, 126.4 (2C), 79.63, 55.4, 49.5, 24.6, 19.2; HRMS *m*/*z* [M + H]^+^ 209.1288 (calcd for C_11_H_17_N_2_O_2_, 209.1290).

*(S)-1-Cyclohexyl-2-nitro-N-[(R)-1-phenylethyl]ethanamine (***7h***, Procedure A).* Pale yellow oil; (0.153 g, 55.3%); separated by HPLC (eluent: hexane/ethyl acetate = 85:25); [α]_D_: +13.8 (*c* = 40 g/100 mL, CHCl_3_); IR ν_max_ 3361, 1558 cm^−1^; ^1^H-NMR (CDCl_3_, 300 MHz) δ 7.38–7.20 (5H, m), 4.28 (1H, dd, *J* = 11.6, 5.1 Hz), 4.19 (1H, dd, *J* = 11.6, 8.0 Hz), 3.87 (1H, q, *J* = 6.5 Hz), 3.07–2.99 (1H, m), 1.86–0.87 (12H, m), 1.31 (3H, d, *J* = 6.5 Hz); ^13^C-NMR (CDCl_3_, 75 MHz) δ 145.0, 128.4 (2C), 127.2, 126.7 (2C), 77.6, 58.8, 55.5, 39.3, 29.2, 28.3, 26.4, 26.3 (2C), 24.6; HRMS *m*/*z* [M + H]^+^ 277.1923 (calcd for C_16_H_25_N_2_O_2_, 277.1916).

*(R)-1-Cyclohexyl-2-nitro-N-[(R)-1-phenylethyl]ethanamine (***7′h***, Procedure B).* Pale yellow oil; (0.065 g, 23.7%); purified by flash chromatography on silica gel (eluent: hexane/ethyl acetate = 85:25); [α]_D_: +18.5 (*c* = 40 g/100 mL, CHCl_3_); IR ν_max_ 3361, 1558 cm^−1^; ^1^H-NMR (CDCl_3_, 300 MHz) δ 7.37–7.19 (5H, m), 4.51 (1H, dd, *J* = 11.6, 5.0 Hz), 4.40 (1H, dd, *J* = 11.5, 5.6 Hz), 3.88 (1H, q, *J* = 6.5 Hz), 2.86 (1H, q, *J* = 5.5 Hz), 1.32 (3H, d, *J* = 6.5 Hz), 1.97–0.81 (12H, m); ^13^C -NMR (CDCl_3_, 75 MHz) δ 145.1, 128.4 (2C), 127.2, 126.8 (2C), 76.1, 58.8, 55.5, 40.2, 29.3, 29.0, 26.3, 26.1 (2C), 24.7; HRMS *m*/*z* [M + H]^+^ 277.1913 (calcd for C_16_H_25_N_2_O_2_, 277.1916).

*(S)-3,3-dimethyl-1-nitro-N-[(R)-1-phenylethyl]butan-2-amine (***7i***,* *Procedure C)*. Pale yellow oil; (0.063 g, 25%); separated by HPLC (eluent: hexane/ethyl acetate = 85:25); [α]_D_: −85.4 (*c* = 40 g/100mL, CHCl_3_); ^1^H-NMR (CDCl_3_, 300 MHz) δ 7.29–7.18 (5H, m), 4.33 (1H, dd, *J* = 11.9, 4.6 Hz), 4.06 (1H, dd, *J* = 11.9, 7.5 Hz), 3.75 (1H, q, *J* = 6.5 Hz), 3.05 (1H, dd, *J* = 7.5 Hz, 4.6 Hz), 1.25 (3H, d, *J* = 6.5 Hz), 1.20 (1H, br, *J* = 2.1 Hz), 0.90 (9H, s); ^13^C NMR (CDCl_3_, 75 MHz) δ 145.7, 128.5 (2C), 127.4, 126.9 (2C), 78.3, 63.2, 57.4, 35.7, 26.7 (3C), 23.6; HRMS *m*/*z* [M + H]^+^ 251.1765 (calcd for C_14_H_23_N_2_O_2_, 251.1760).

*(R)-3,3-dimethyl-1-nitro-N-[(R)-1-phenylethyl]butan-2-amine (***7′i***,* *Procedure D).* Pale yellow oil; (0.138 g, 55%); purified by flash chromatography on silica gel (eluent: hexane/ethyl acetate = 85:25); [α]_D_: −43.6 (*c* = 40 g/100 mL, CHCl_3_); IR ν_max_ 3359 1553 cm^−1^; ^1^H-NMR (CDCl_3_, 300 MHz) δ 7.33–7.24 (5H, m), 4.59 (1H, dd, *J* = 11.8, 4.5 Hz), 4.34 (1H, dd, *J* = 11.7, 6.4 Hz), 3.82 (1H, q, *J* = 6.5 Hz), 2.86 (1H, dd, *J* = 6.3 Hz, 4.6 Hz), 1.59 (1H, br), 1.32 (3H, d, *J* = 6.5 Hz), 0.85 (9H, s); ^13^C-NMR (CDCl_3_, 75 MHz) δ 144.9, 128.3 (2C), 127.2 (2C), 127.1, 77.5, 62.7, 56.3, 35.1, 26.4 (3C), 24.5; HRMS *m*/*z* [M + H]^+^ 251.1763 (calcd for C_14_H_23_N_2_O_2_, 251.1760).

*3-Nitro-N-[(R)-1-phenylethyl]butan-2-amine (syn-***8g**/**8′g***;anti-***9g**/**9′g***, Procedure A)* Yellow oil; (0.186 g, 84%); purified by flash chromatography on silica gel (eluent: hexane/ethyl acetate = 80:20); IR ν_max_ 3355, 1567 cm^−1^; ^1^H -NMR (CDCl_3_, 300 MHz) δ 7.48–7.07 (20H, m), 4.76–4.62 (2H, m), 4.56–4.30 (2H, m), 3.99–3.86 (3H, m), 3.87–3.70 (1H, m), 3.24–3.08 (1H, m), 3.03–2.83 (3H, m), 1.54–1.35 (4H, m), 1.48 (3H, d, *J* = 7.0 Hz), 1.44 (6H, d, *J* = 6.6 Hz), 1.40 (3H, d, *J* = 6.4 Hz), 1.33 (9H, d, *J* = 6.4 Hz), 1.30 (3H, d, *J* = 6.5 Hz), 1.12 (3H, d, *J* = 6.5 Hz), 1.11 (3H, d, *J* = 6.4 Hz), 1.01 (3H, d, *J* = 6.7 Hz), 0.98 (3H, d, *J* = 6.8 Hz); ^13^C-NMR (CDCl_3_, 75 MHz) δ 145.5, 145.2, 144.7, 144.6, 128.4 (4C), 128.3, 128.2 (4C), 126.9 (4C), 126.6, 126.5, 126.3 (4C), 126.2, 87.8, 87.3, 87.0, 84.4, 55.7, 55.1, 54.9, 53.8 (2C), 53.7, 53.1, 52.9, 25.1, 24.7, 24.4, 23.7, 16.6, 16.5 (2C), 15.8, 15.6, 15.3, 14.5 (2C); HRMS *m*/*z* [M + H]^+^ 223.1450 (calcd for C_12_H_19_N_2_O_2_, 223.1447). 

*(2R,3S)-3-Nitro-N-[(R)-1-phenylethyl]butan-2-amine (anti-**9*****′*g****, Procedure B).* Yellow oil; (0.133 g, 60%); purified by flash chromatography on silica gel (eluent: hexane/ethyl acetate = 80:20); [α]_D_: −9.9 (*c* = 40 g/100 mL, CHCl_3_); IR ν_max_ 3353, 1566 cm^−1^; ^1^H-NMR (CDCl_3_, 300 MHz) δ 7.37–7.22 (5H, m), 4.75–4.63 (1H, m), 3.94 (1H, q, *J* = 6.5 Hz), 2.95 (1H, m), 2.00 (1H, br), 1.44 (3H, d, *J* = 6.7 Hz), 1.32 (3H, d, *J* = 6.5 Hz), 1.00 (3H, d, *J* = 6.6 Hz); ^13^C-NMR (CDCl_3_, 75 MHz) δ 145.3, 128.5 (2C), 127.1, 126.4 (2C), 84.5, 55.2, 54.0, 24.5, 16.7, 14.7; HRMS *m*/*z* [M + H]^+^ 223.1445 (calcd for C_12_H_19_N_2_O_2_, 223.1447).

*(R)-1-Cyclohexyl-2-nitro-N-[(R)-1-phenylethyl]propan-1-amine (syn-***8'h***/anti-***9'h***,* *Procedure B).* Pale yellow oil; (0.162 g, 56%); purified by flash chromatography on silica gel (eluent: hexane/ethyl acetate = 80:20); IR ν_max_ 3357, 1568 cm^−1^; ^1^H-NMR (CDCl_3_, 300 MHz) δ 7.36–7.21 (10H, m), 4.62 (1H, q, *J* = 6.9 Hz, *syn*), 4.25 (1H, q, *J* = 6.5 Hz, *anti*), 3.76 (1H, q, *J* = 6.8 Hz, *anti*), 3.74 (1H, q, *J* = 6.2 Hz, *syn*), 2.93 (1H, dd, *J* = 5.9 Hz, 4.2 Hz, *anti*), 2.83 (1H, dd, *J* = 7.5 Hz, 4.1 Hz, *syn*), 1.52 (3H, d, *J* = 3.9 Hz, *syn*), 1.48 (3H, d, *J* = 6.8 Hz, *anti*), 1.83–0.94 (24H, m), 1.27 (6H, d, *J* = 6.5 Hz); ^13^C NMR (CDCl_3_, 75 MHz) δ 145.4, 145.2, 128.4 (2C), 128.3 (2C), 127.2, 127.1, 126.9 (2C), 126.5 (2C), 86.6, 83.8, 69.4, 62.8, 57.0, 56.7, 41.1, 40.2, 30.8, 30.7, 29.8 (2C), 26.1 (2C), 25.4 (2C), 24.7, 23.9, 21.7, 21.4, 16.8, 13.5; HRMS *m*/*z* [M + H]^+^ 291.2074 (calcd for C_17_H_27_N_2_O_2_ 291.2073).

## 4. Conclusions

In summary, a first direct comparison in aza-Henry addition reactions between the *C*-CF_3_ and *C*-CH_3_ substituted *N*-protected aldimines was reported. The different inductive effect of the two groups greatly influence the reaction outcome acting both on the electrophilicity of the imino carbon and on the nitrogen lone pair availability. The presence of a strong steric hindrance on the imine carbon due to the *tert*-butyl group unexpectedly required the reaction conditions already fixed for trifluoromethyl aldimines, although for different reactivity reasons.

## Figures and Tables

**Table 1:** Solvent-free synthesis of *C*-alkyl substituted *N*-protected aldimines. 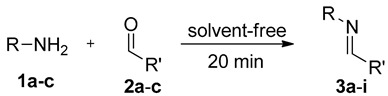

Entry	1	R	2	R′	Product 3	Yield (%)
1	**a**	Bn	**a**	Me	**a**	85
2			**b**	Cy	**b**	90
3			**b**	*t-*Bu	**c**	95
4	**b**	Ph	**a**	Me	**d**	78
5			**b**	Cy	**e**	80
6			**c**	*t-*Bu	**f**	83
7	**c**	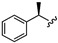	**a**	Me	**g**	80
8			**b**	Cy	**h**	88
9			**c**	*t-*Bu	**i**	95

**Table 2:** Stereoselective comparison between the aza-Henry additions on *C*-alkyl substituted aldimines. 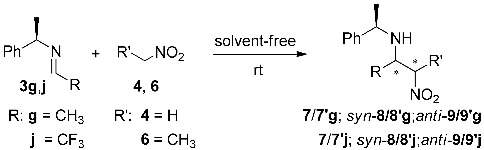

Entry	R	R′	Product	Time (h)	Yield (%) ^b^	*syn*/*anti* ^a^	Dr ^a^
1	CH_3_	H	**7/7′g**	1	90	−	3:7
2	CF_3_^c^		**7/7′j**	3	80	−	8:2
3	CH_3_	CH_3_	*syn*-**8/8′g**; *anti*-**9/9′g**	8	84	1:1	2:8
4	CF_3_^c^		*syn*-**8/8′j**; *anti*-**9/9′j**	18	70	3:7	7.2:2.8

^a^ Determined by ^1^H-NMR performed on the crude mixtures. ^b^ After flash chromatography on silica gel. ^c^ Reaction performed in the presence of ZrCl_4_ as catalyst, see ref. [10].

**Table 3:** Diastereoselective additions on different *C*-alkyl substituted aldimines. 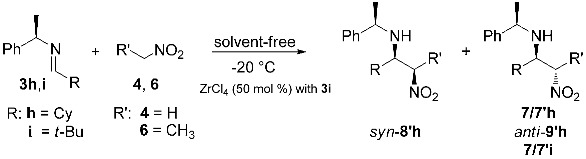

Entry	R	R'	Product	Time (h)	Yield (%) ^b^	*syn*/*anti* ^a^	Dr ^a^
1	Cy	H	**7/7′h**	24	45	−	99:1
2		CH_3_	*syn*-**8/8′h**; *anti*-**9/9′h**	48	56	1:1	99:1
3	*t*-Bu ^c^	H	**7/7′i**	8	55	−	99:1
4		CH_3_	−	−	−	−	−

^a^ Determined by ^1^H-NMR performed on the crude mixtures; ^b^ After purification on silica gel; ^c^ Reaction performed in the presence of ZrCl_4_ (50 mol %).

## Supplementary Materials

Supplementary materials can be accessed at: http://www.mdpi.com/1420-3049/21/6/723/s1.
