# Supplementary material for: Aza-Henry Reactions on C-Alkyl Substituted Aldimines
Source: Molecules. 2016 Jun 2;21(6):723. doi: 10.3390/molecules21060723 (PMC6273577; doi:10.3390/molecules21060723)
Supplement: Supplementary file 1 [file molecules-21-00723-s001.pdf]

Supplementary Materials: Aza-Henry Reactions on *C*-Alkyl Substituted Aldimines

Alessia Pelagalli, Lucio Pellacani, Elia Scandozza and Stefania Fioravanti

^{1}H- and ^{13}C-NMR spectra of all new compounds

N-Benzyl-1-nitropropan-2-amine (5a)

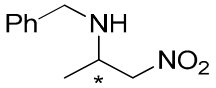

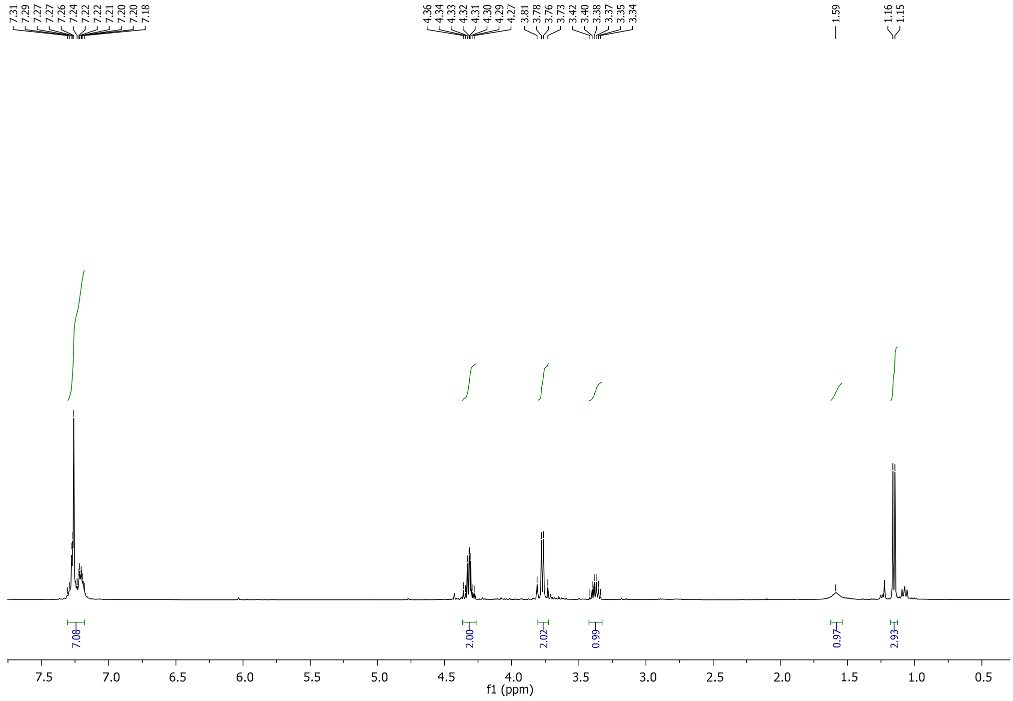

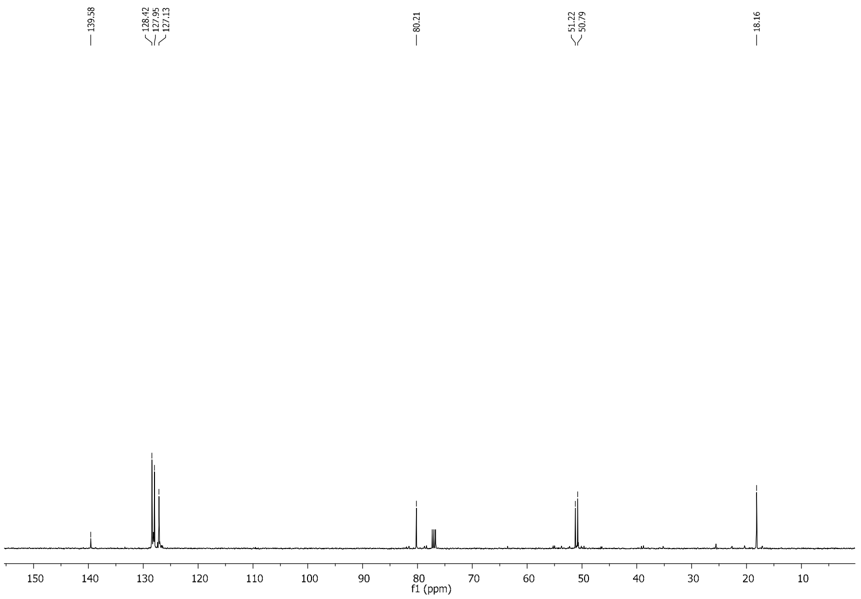

N-Benzyl-1-cyclohexyl-2-nitroethanamine (5b)

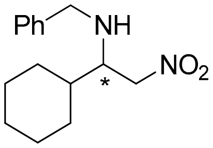

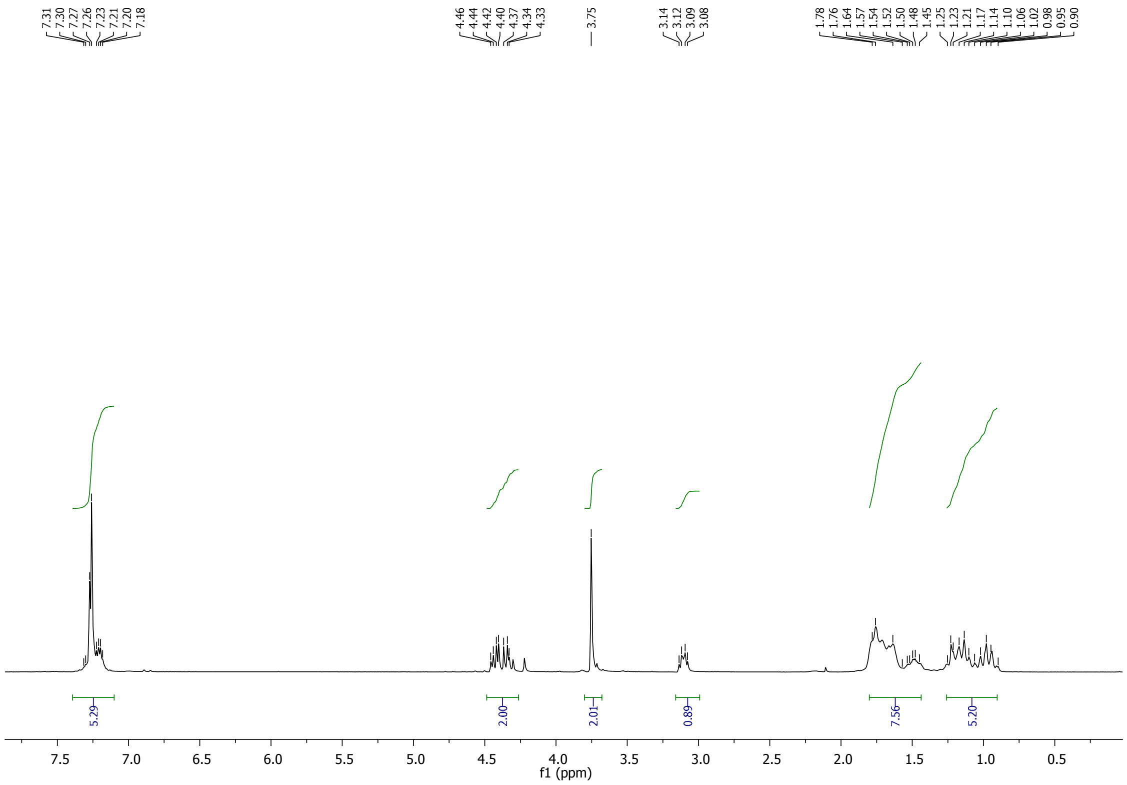

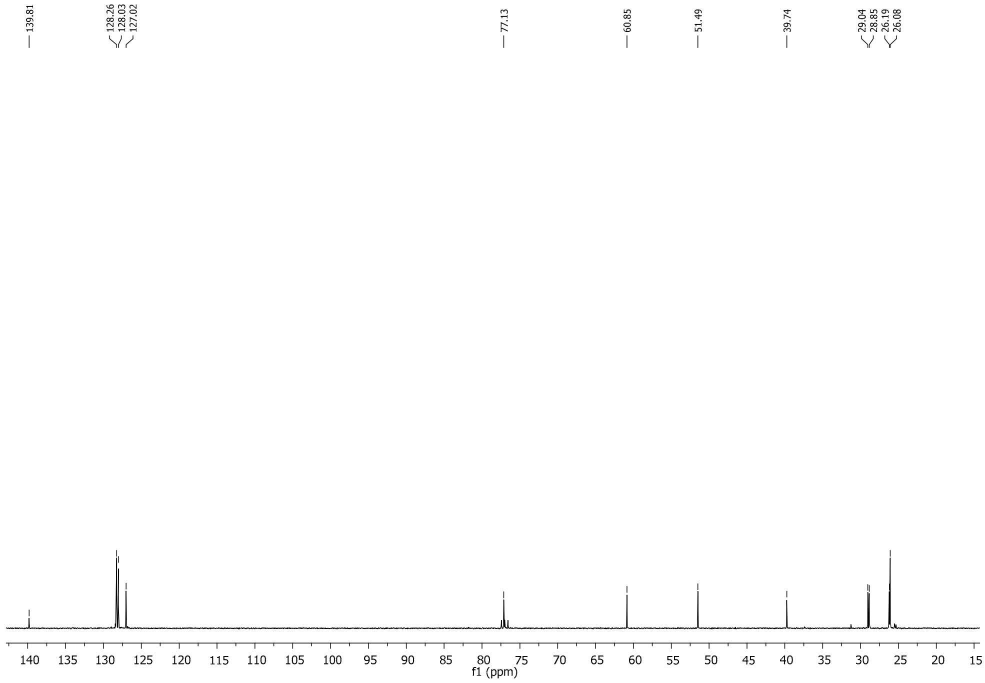

N-Benzyl-3,3-dimethyl-1-nitrobutan-2-amine (5c)

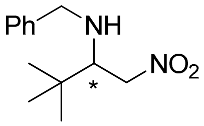

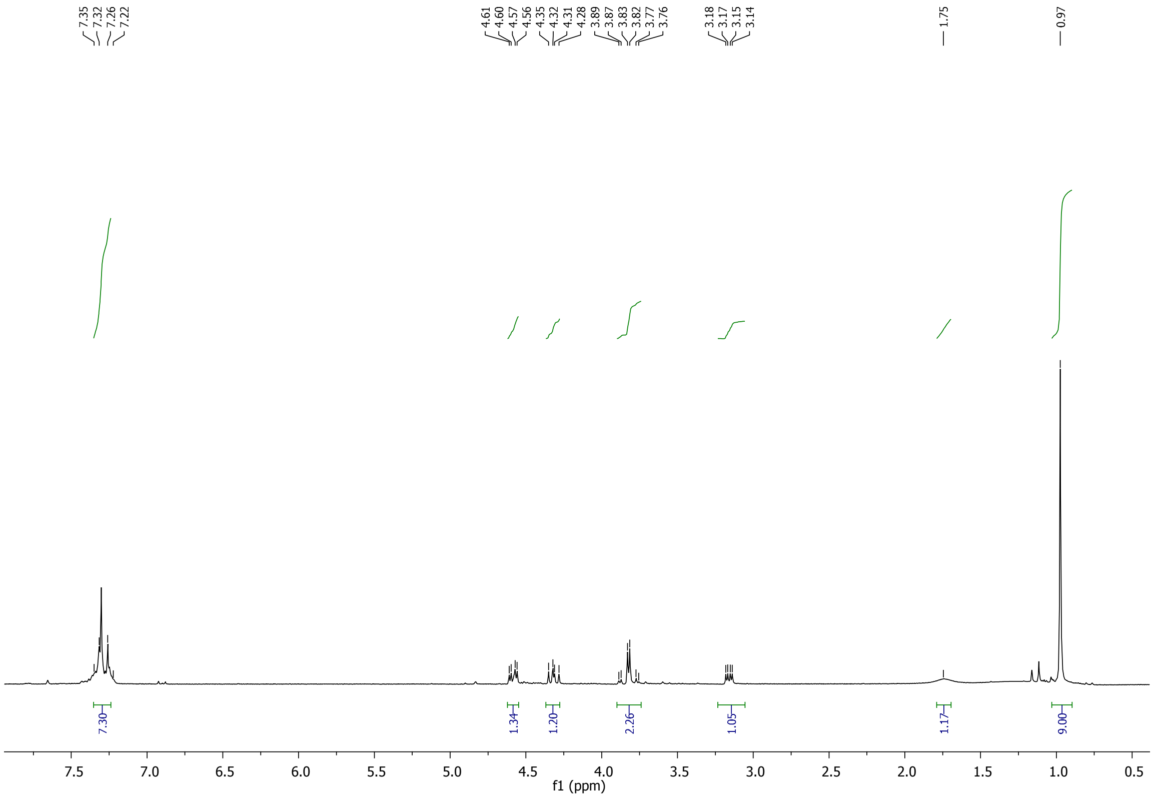

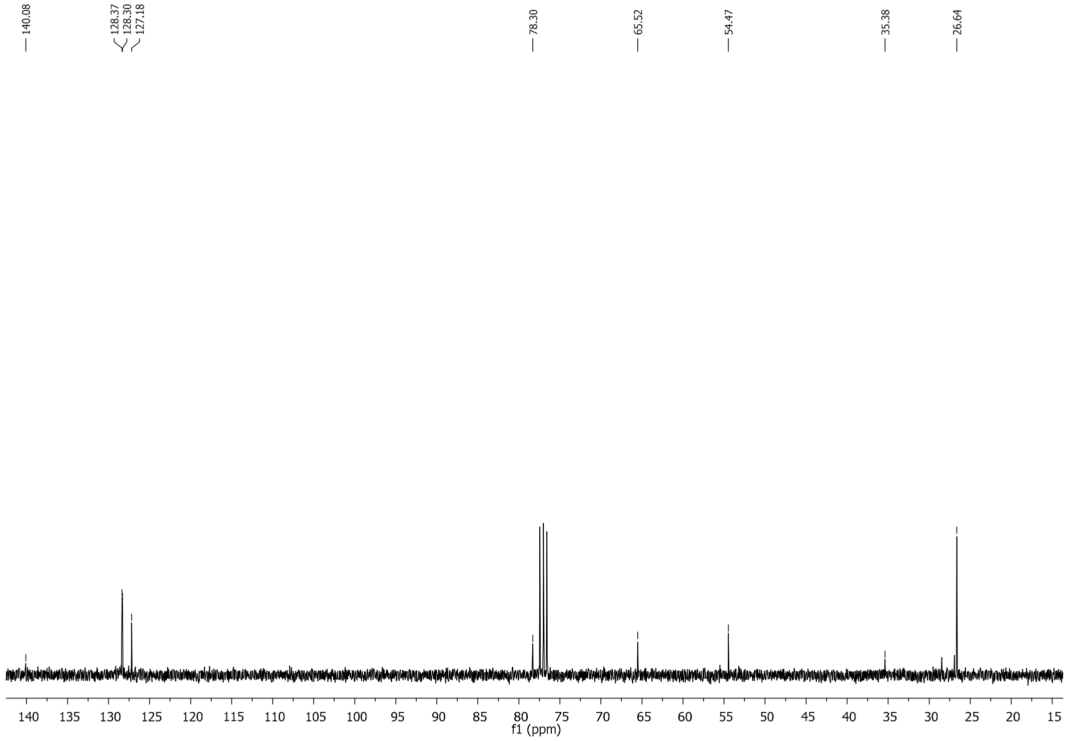

N-(1-Nitropropan-2-yl)aniline (5d)

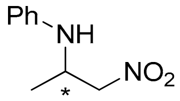

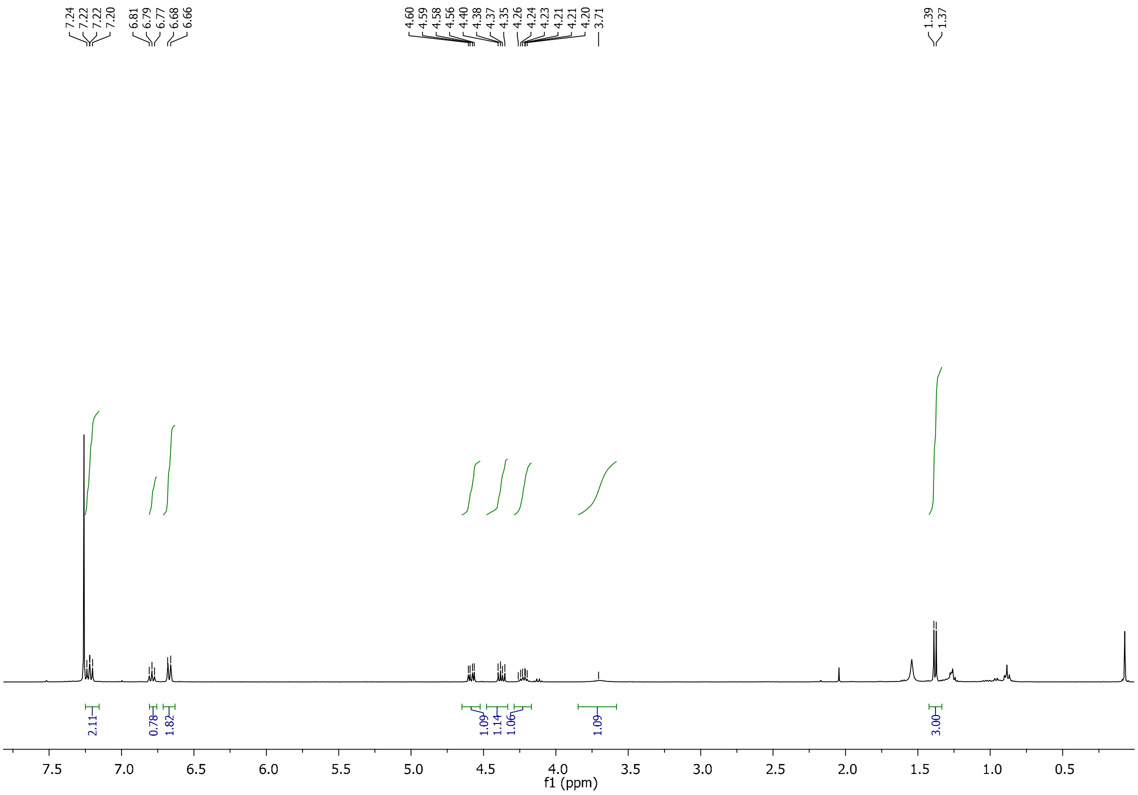

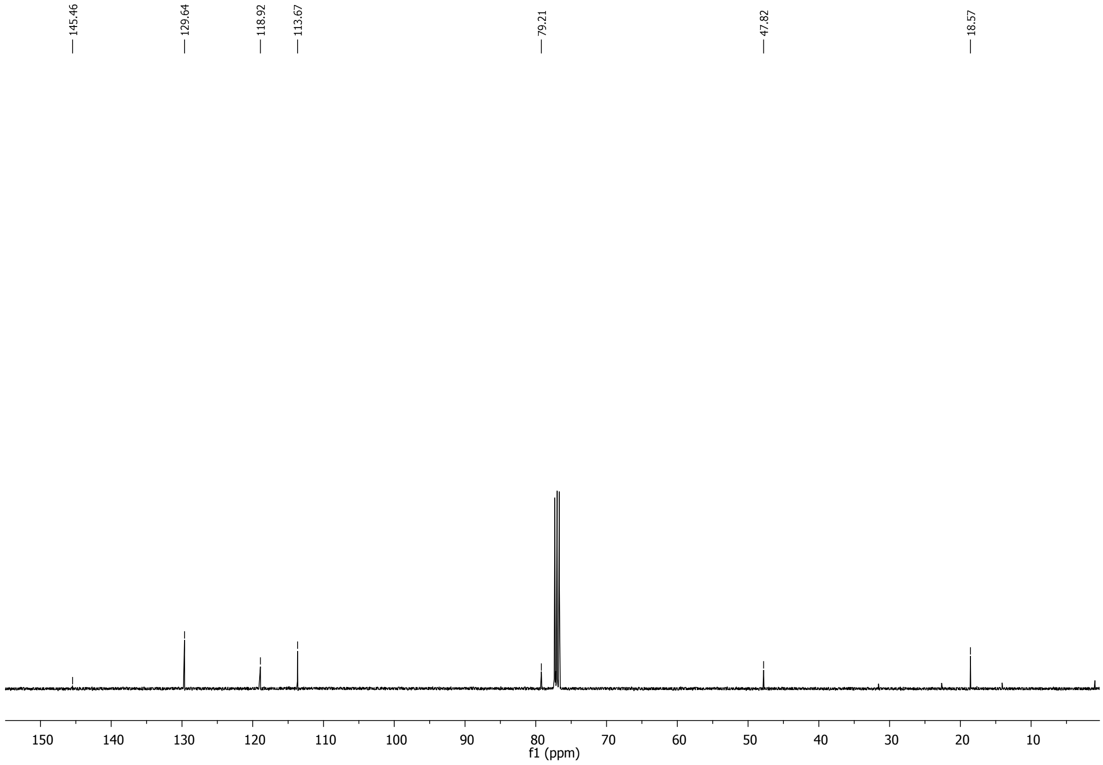

N-(1-Cyclohexyl-2-nitroethyl)aniline (5e)

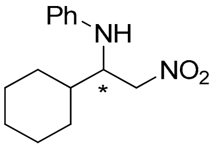

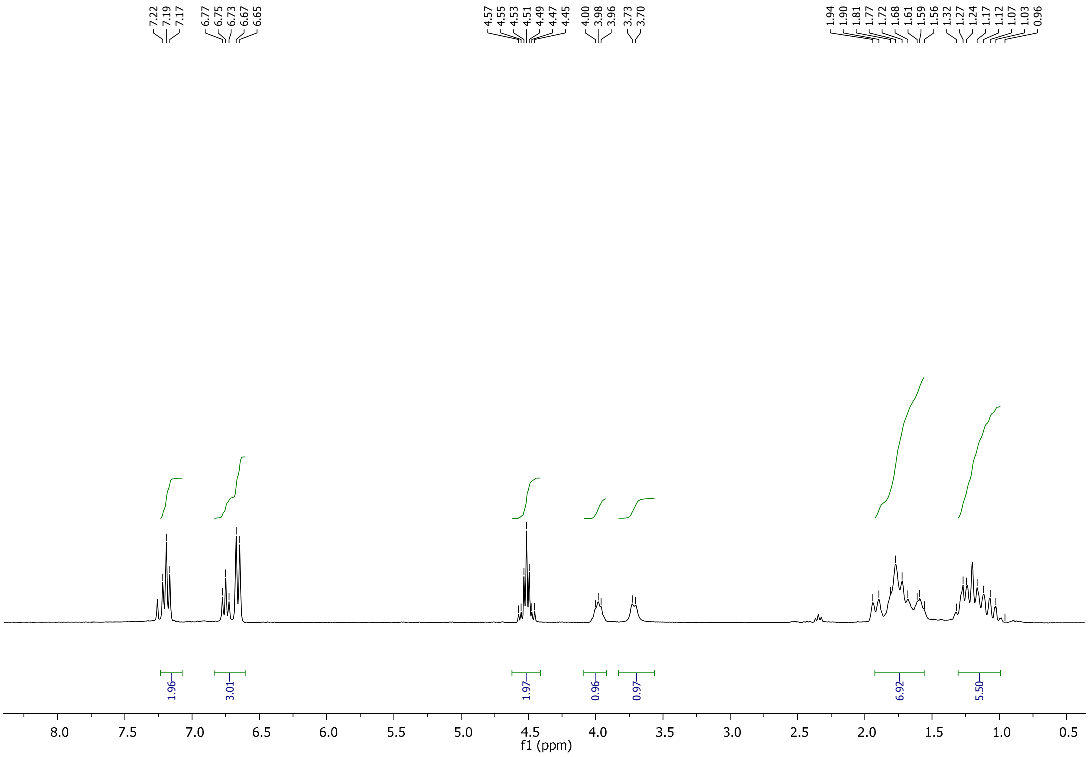

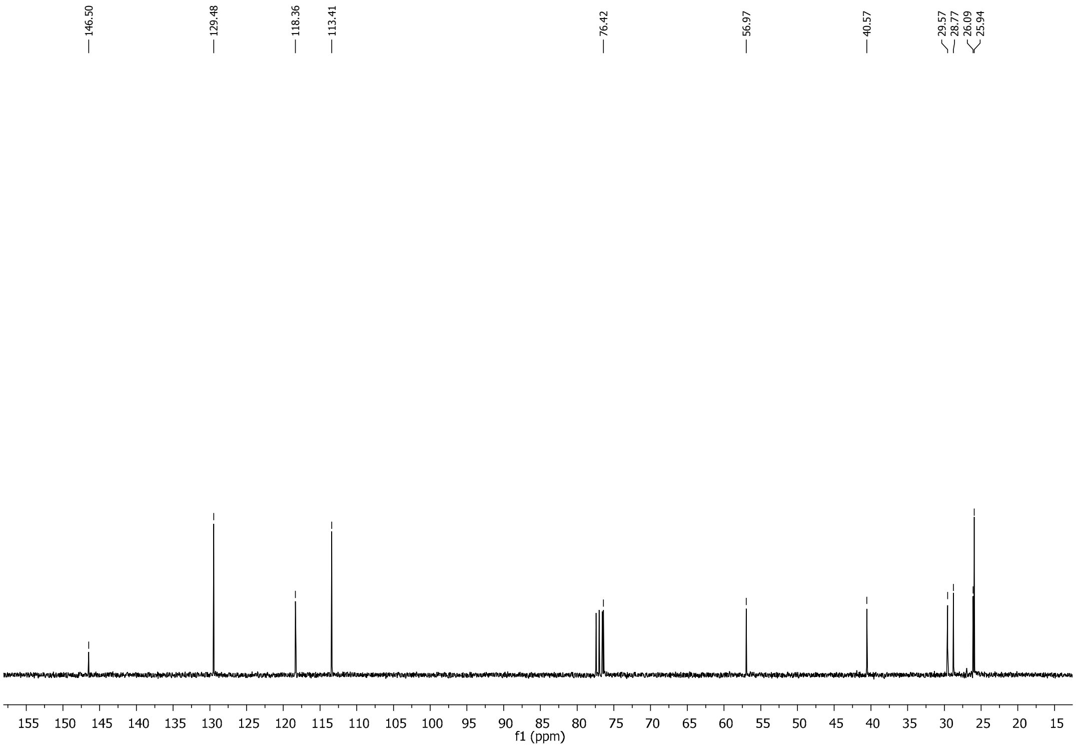

N-(3,3-Dimethyl-1-nitrobutan-2-yl)aniline (5f)

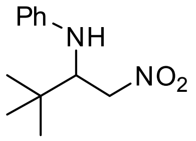

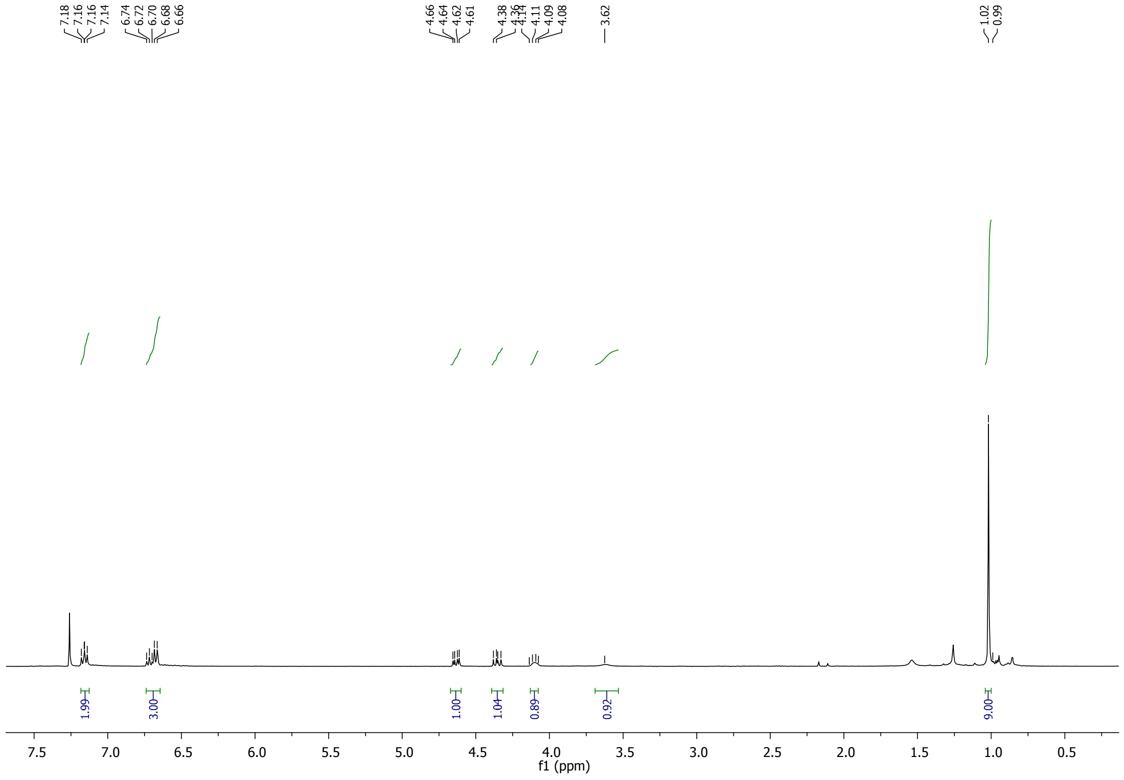

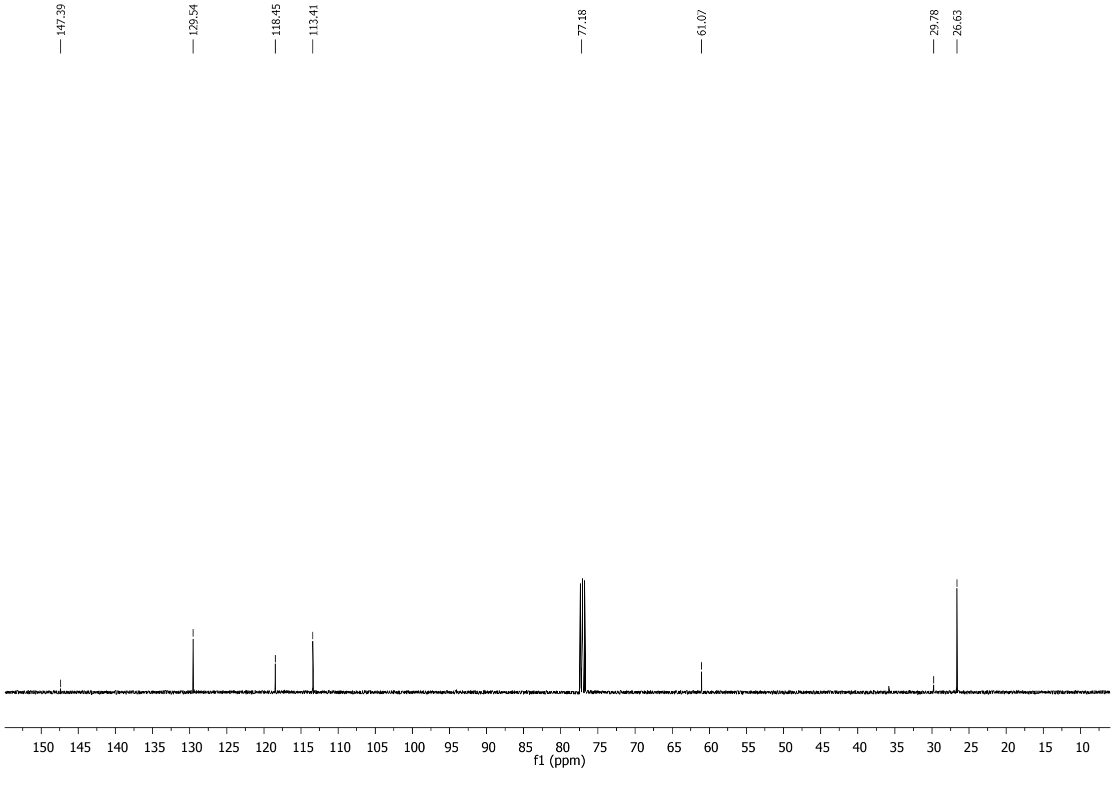

(*S*)-1-Nitro-*N*-[(*R*)-1-phenylethyl]propan-2-amine (7g)

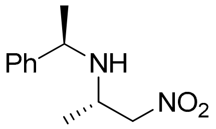

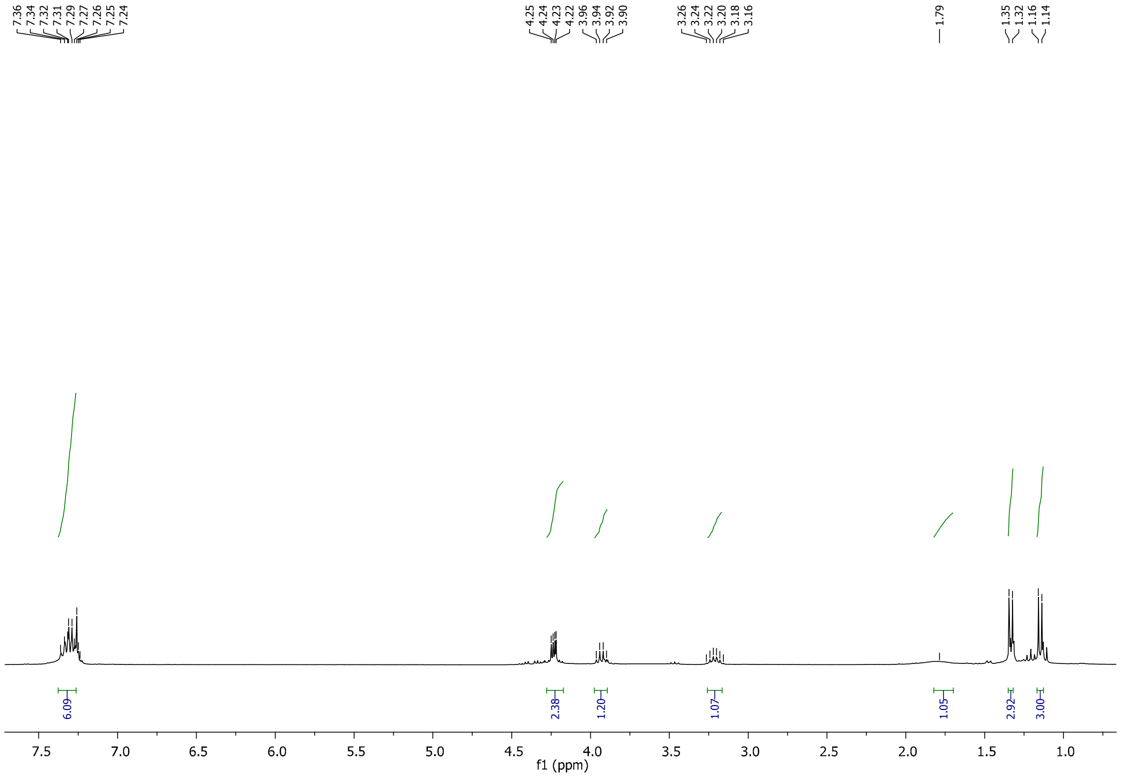

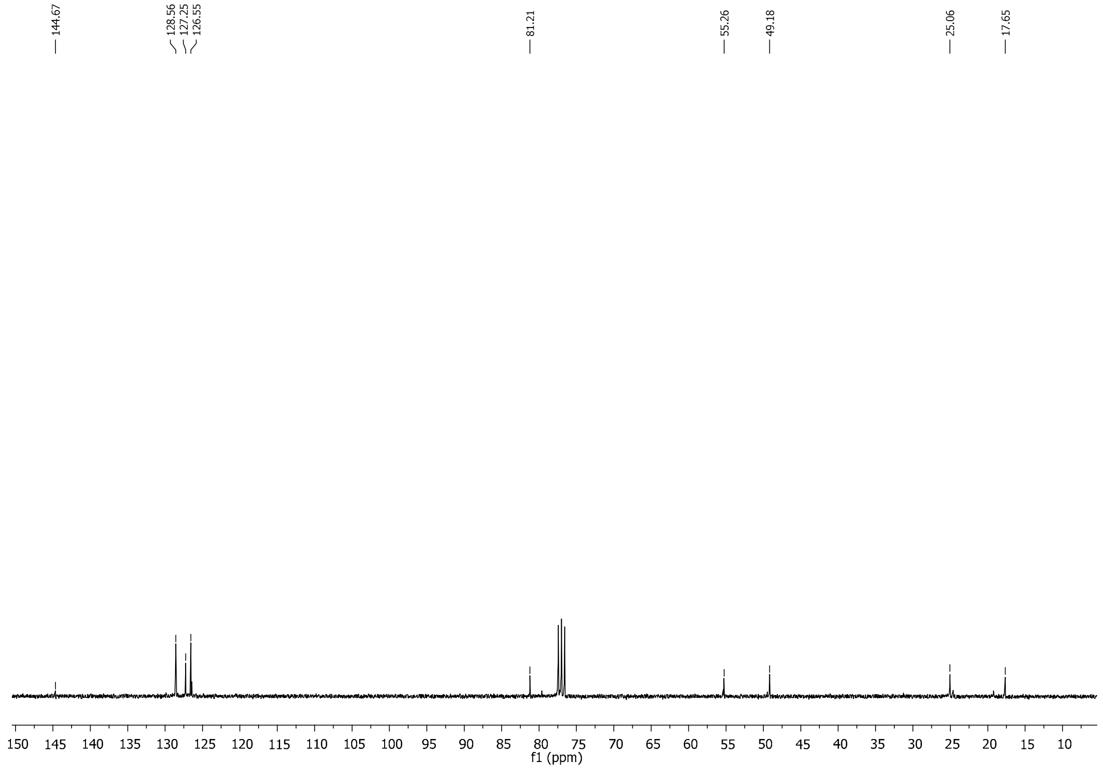

(*R*)-1-Nitro-*N*-[(*R*)-1-phenylethyl]propan-2-amine (7′g)

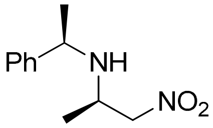

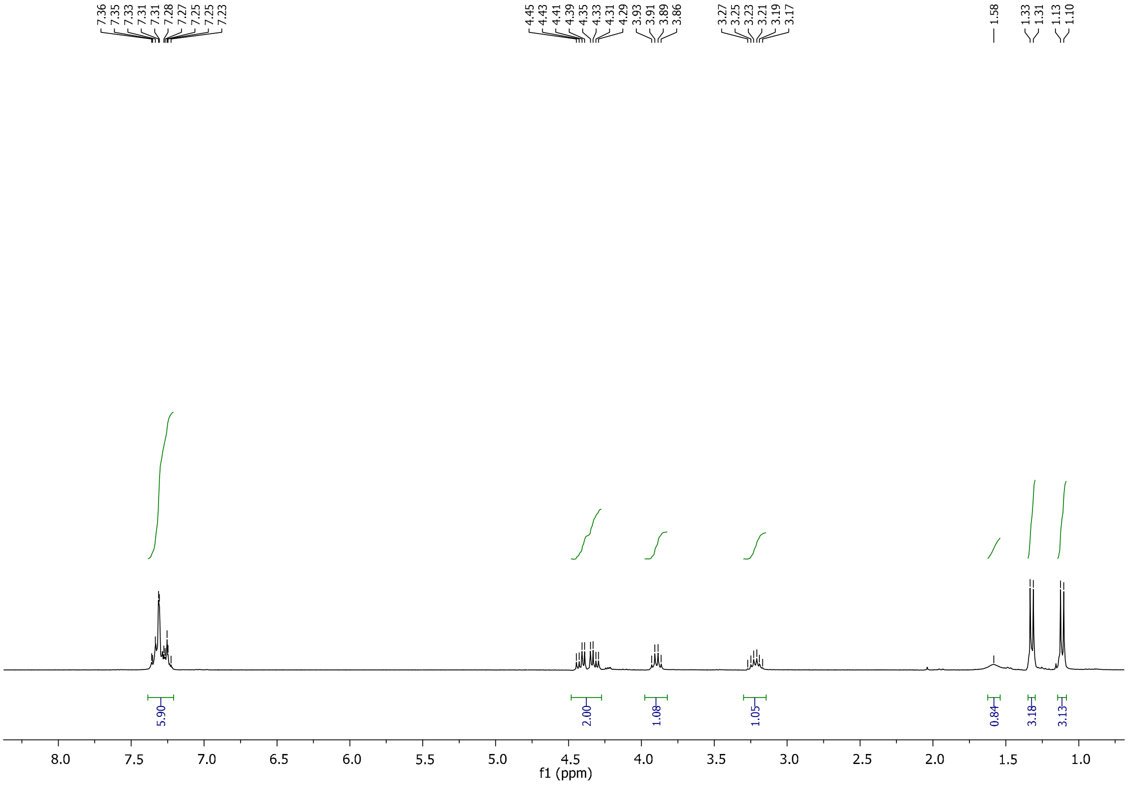

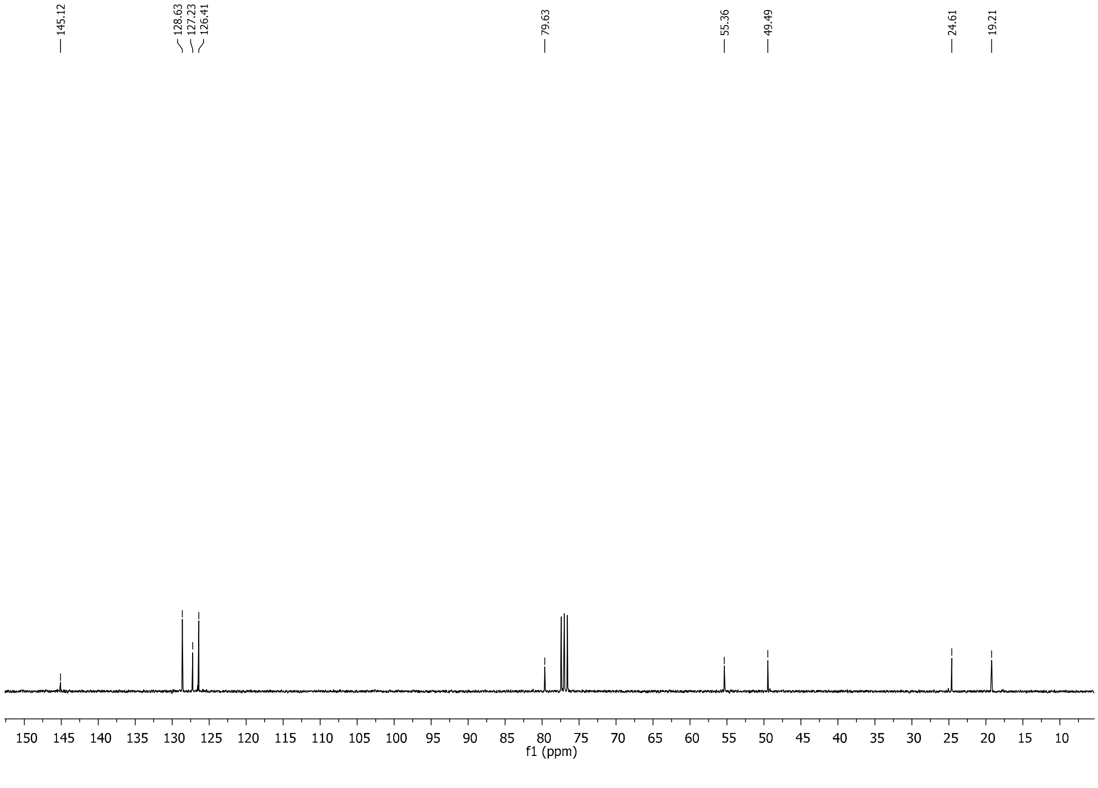

(*S*)-1-Cyclohexyl-2-nitro-*N*-[(*R*)-1-phenylethyl]ethanamine (7h)

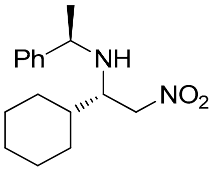

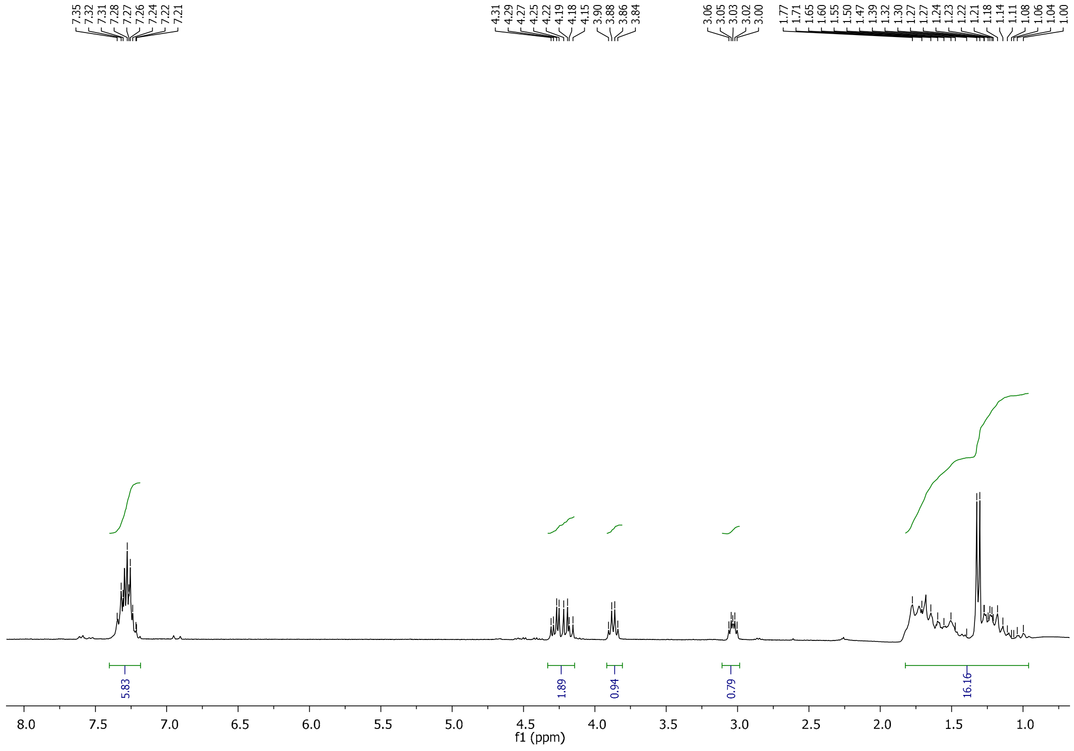

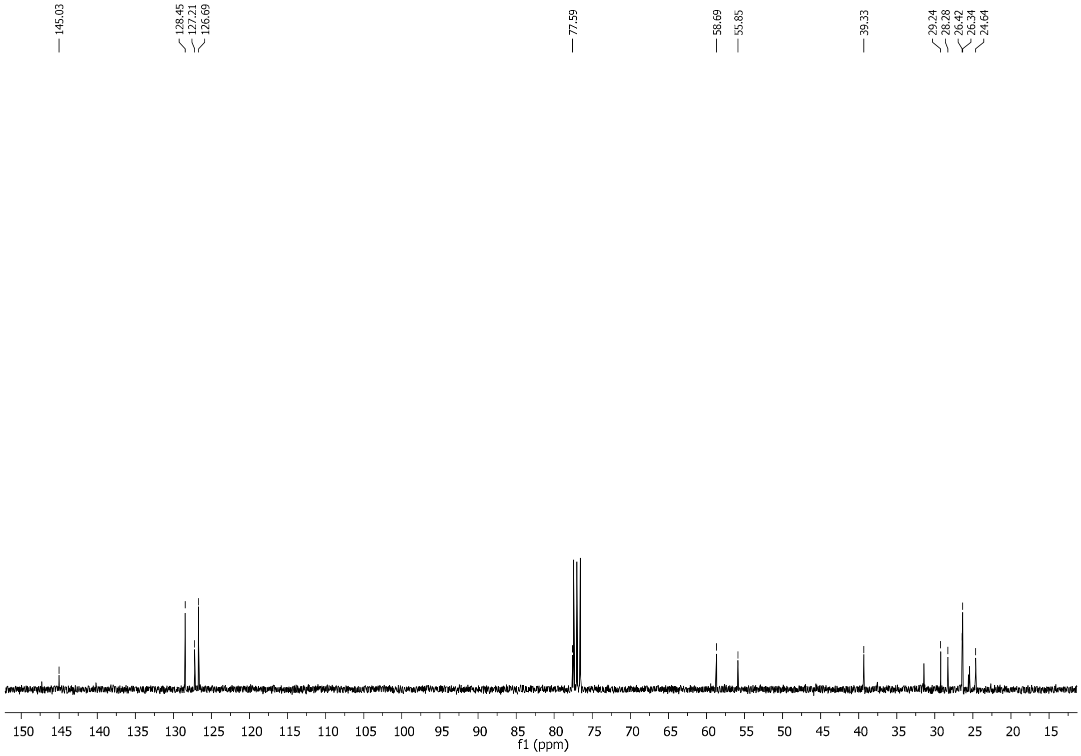

(*R*)-1-Cyclohexyl-2-nitro-*N*-[(*R*)-1-phenylethyl]ethanamine (7'h)

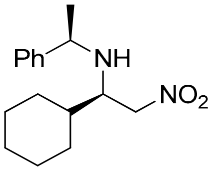

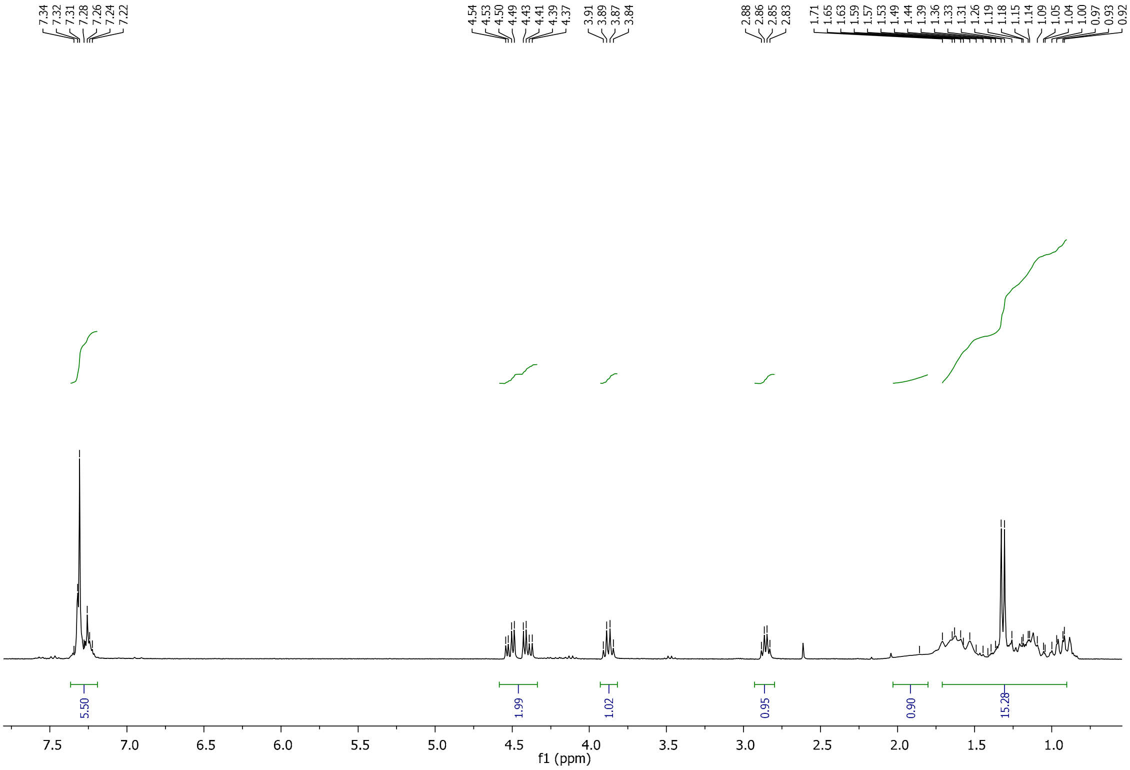

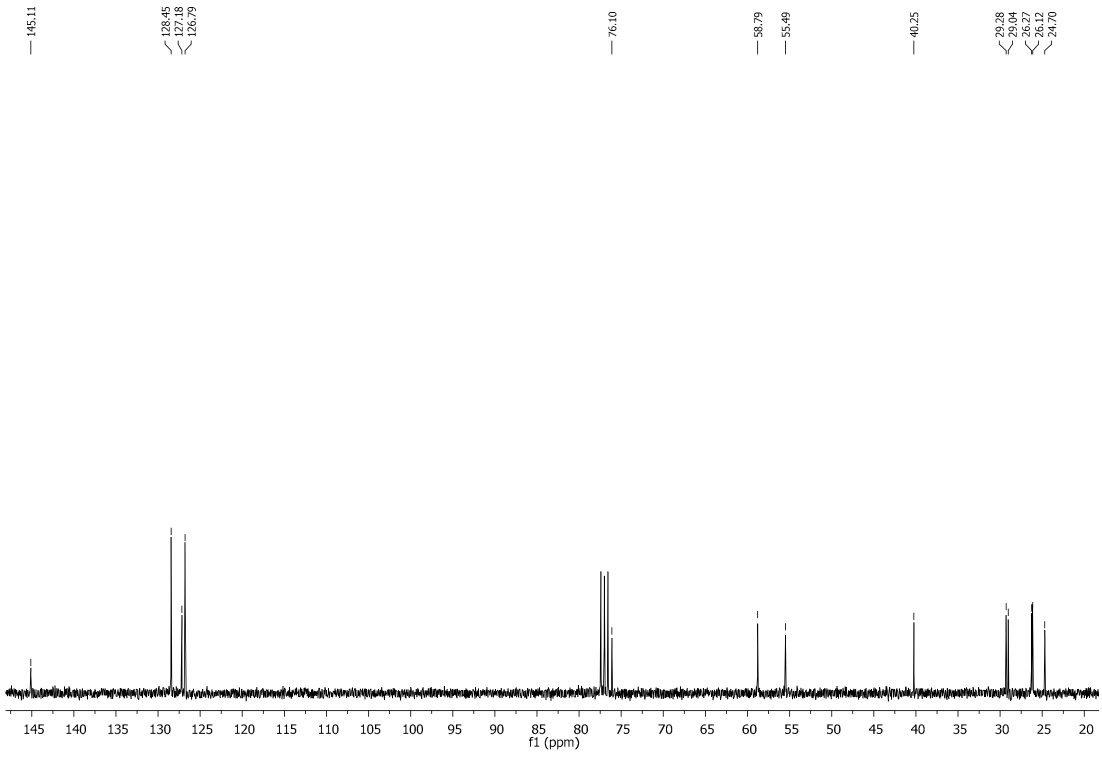

(*S*)-3,3-dimethyl-1-nitro-*N*-[(*R*)-1-phenylethyl]butan-2-amine (7i)

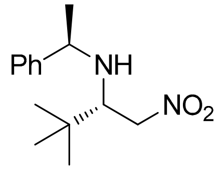

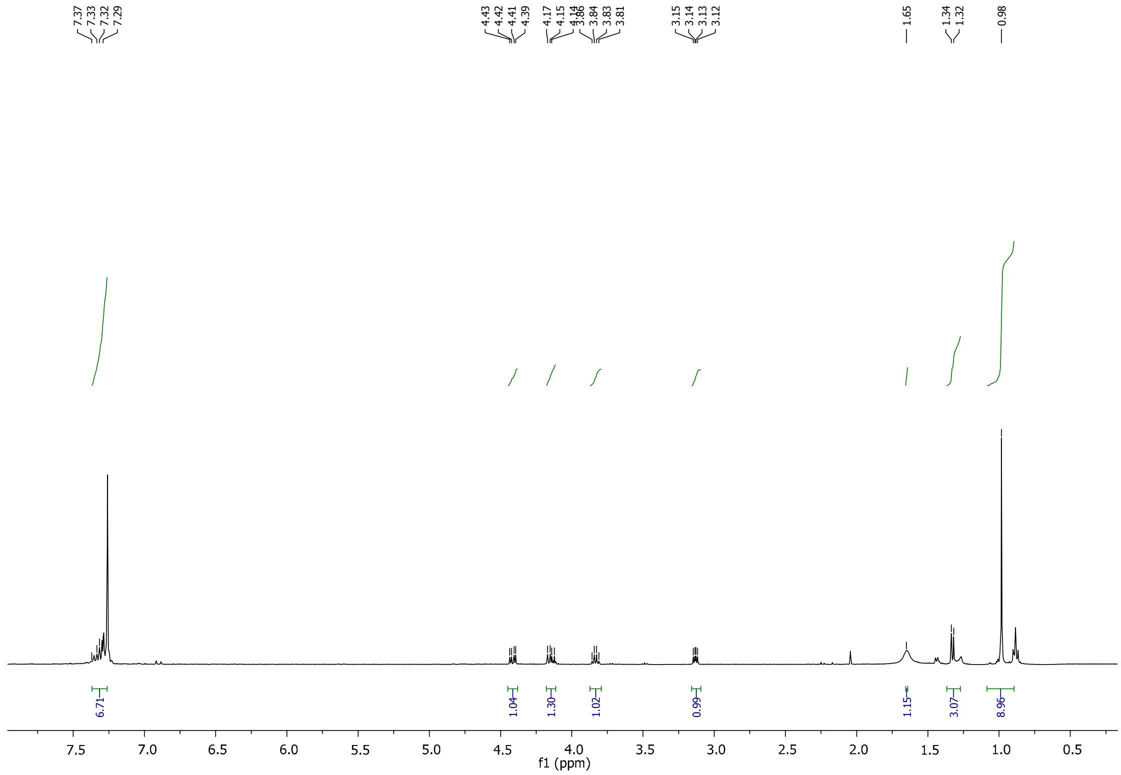

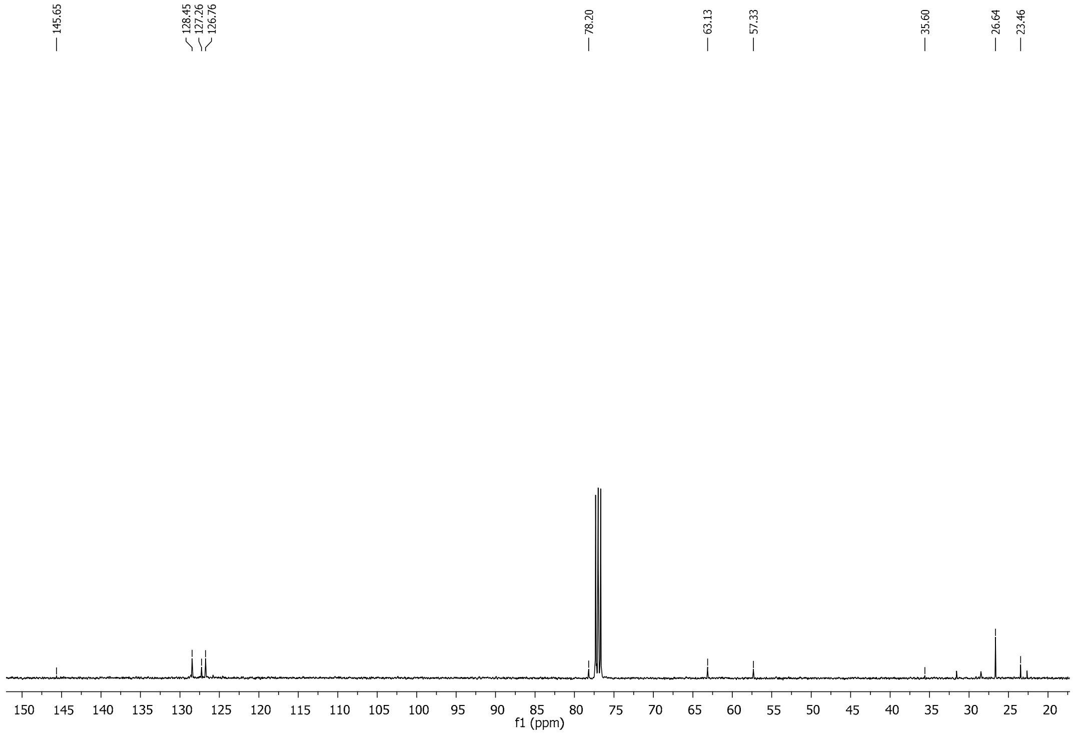

(*R*)-3,3-dimethyl-1-nitro-*N*-[(*R*)-1-phenylethyl]butan-2-amine (7′i)

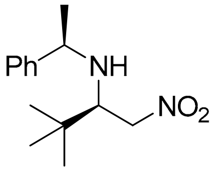

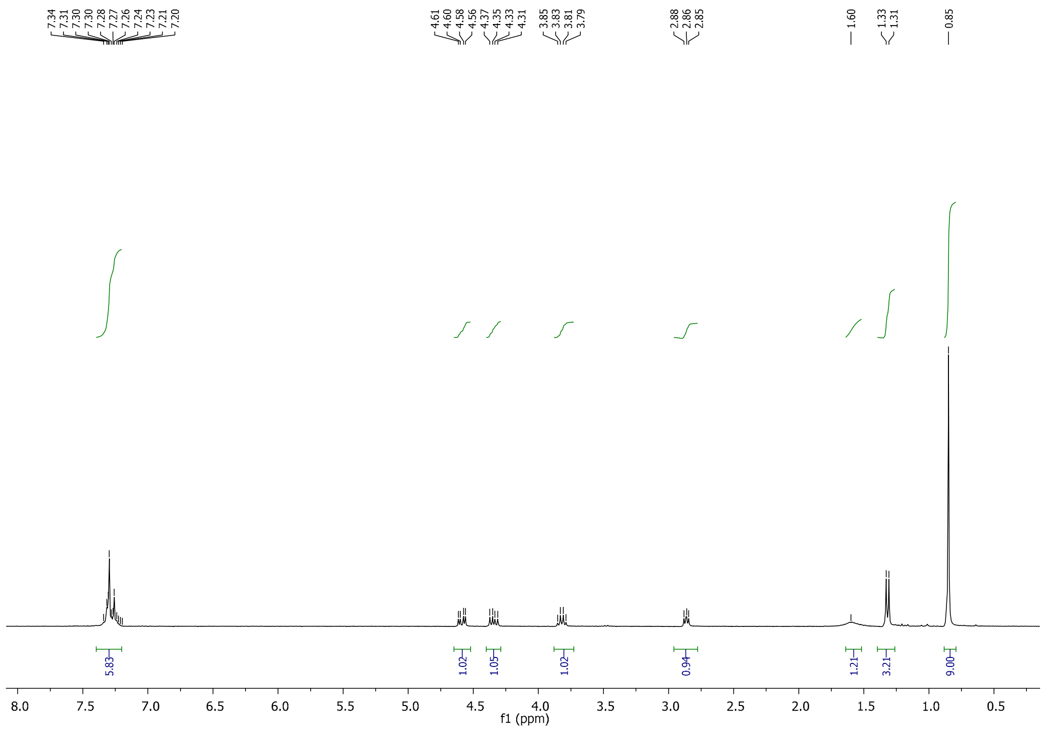

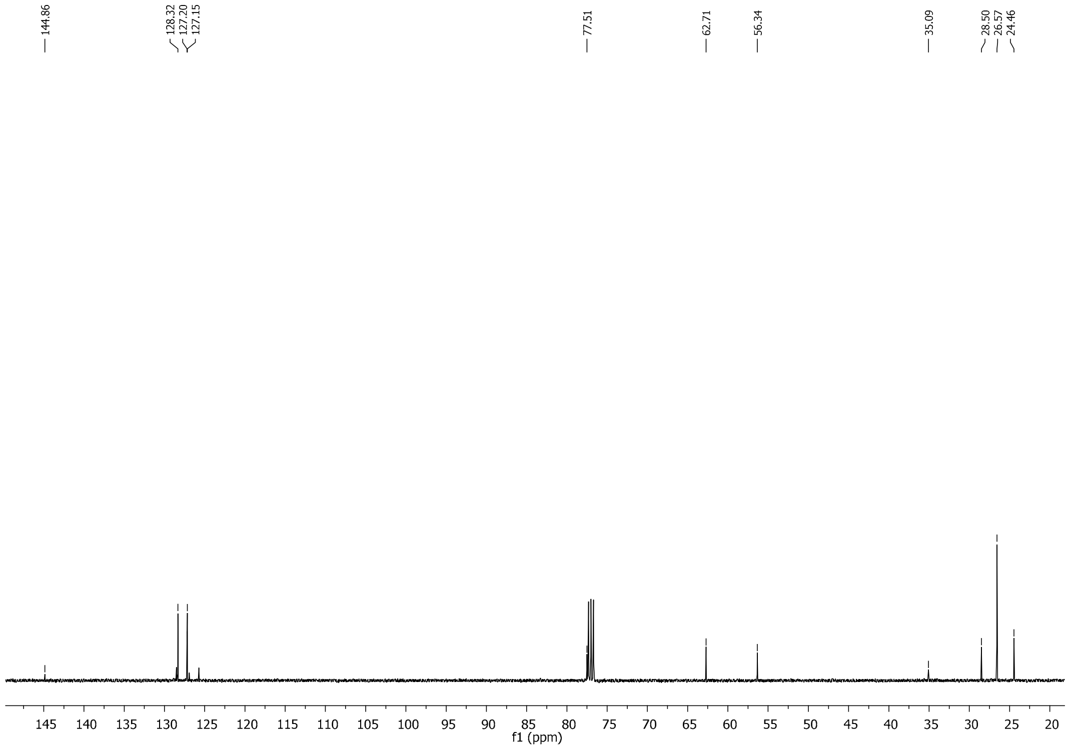

3-Nitro-*N*-[(*R*)-1-phenylethyl]butan-2-amine (*syn*-8/8'g;*anti*-9/9'g)

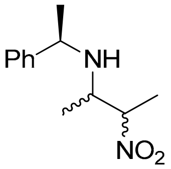

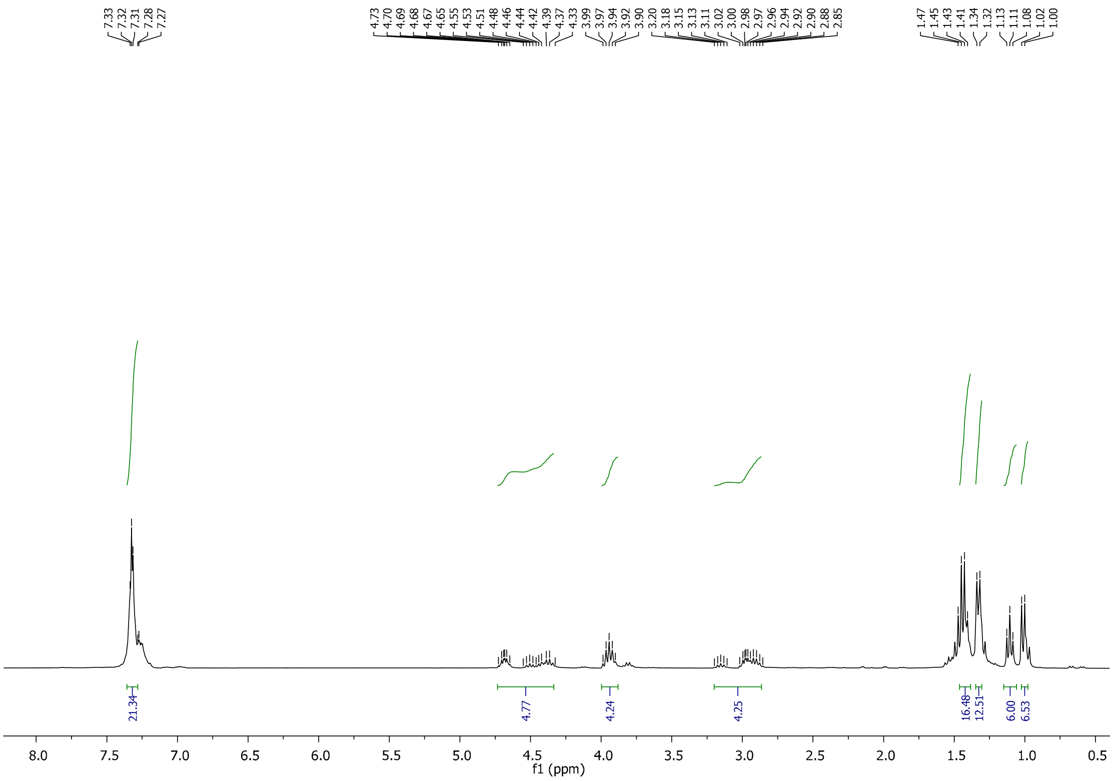

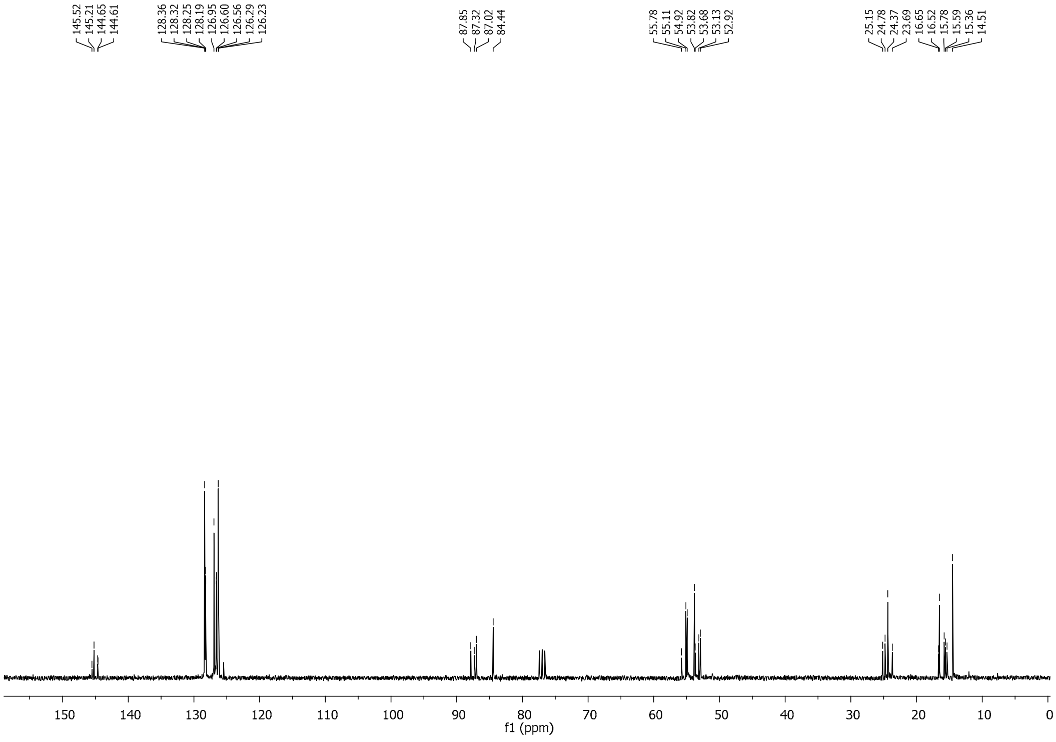

(2*R*,3*S*)-3-Nitro-*N*-[(*R*)-1-phenylethyl]butan-2-amine (*anti*-9'g)

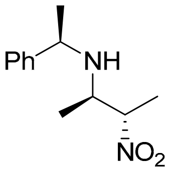

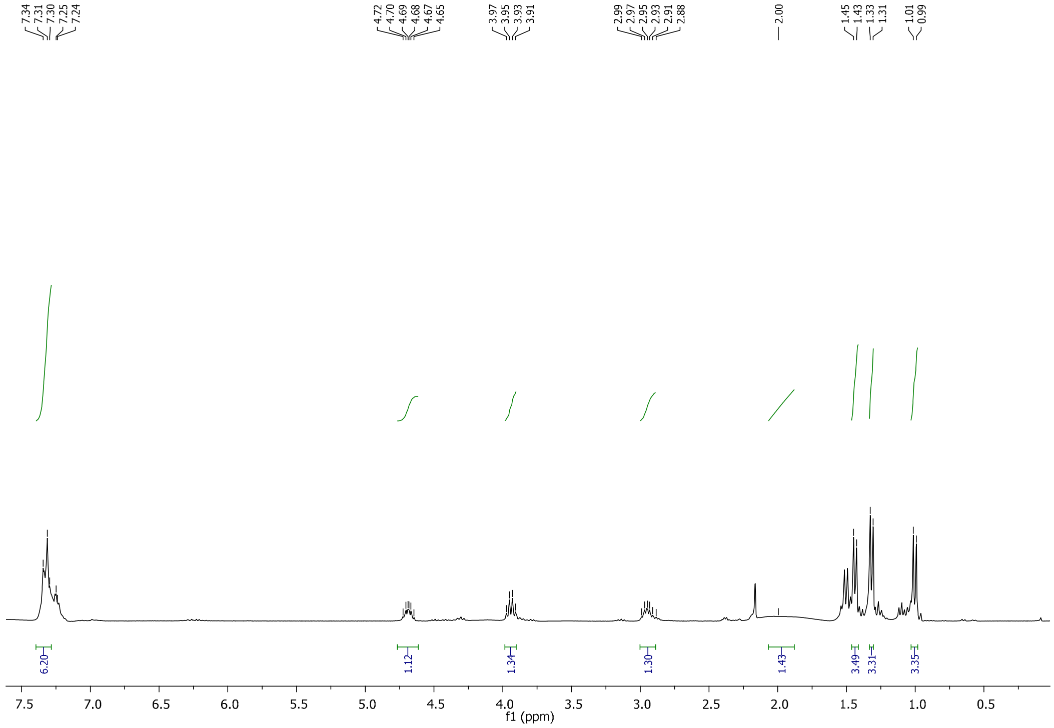

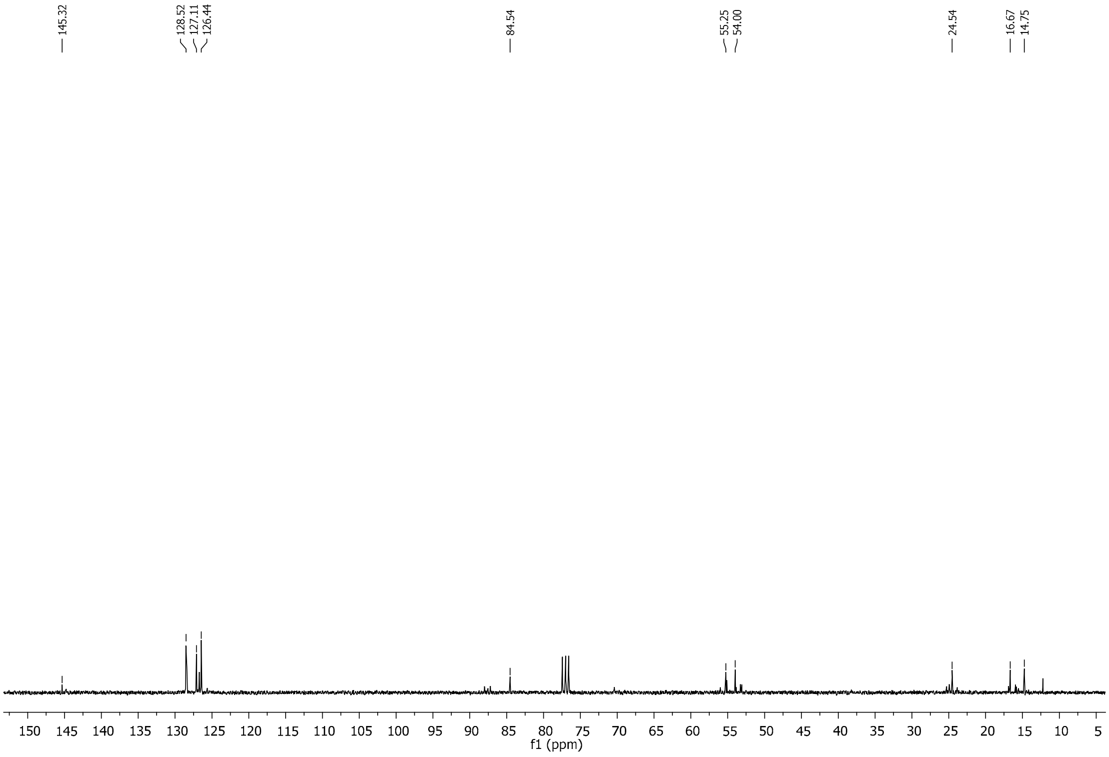

(*R*)-1-Cyclohexyl-2-nitro-*N*-(1-phenylethyl)propan-1-amine (*syn*-8'h/*anti*-9'h)

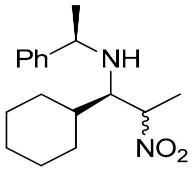

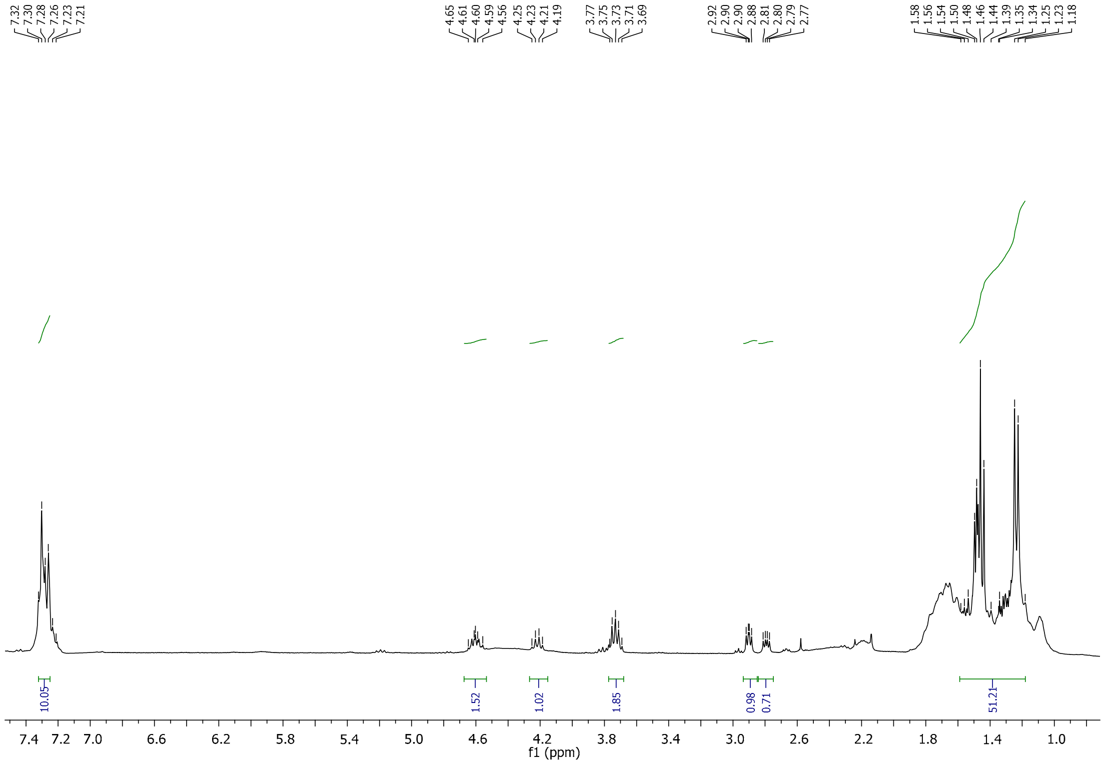

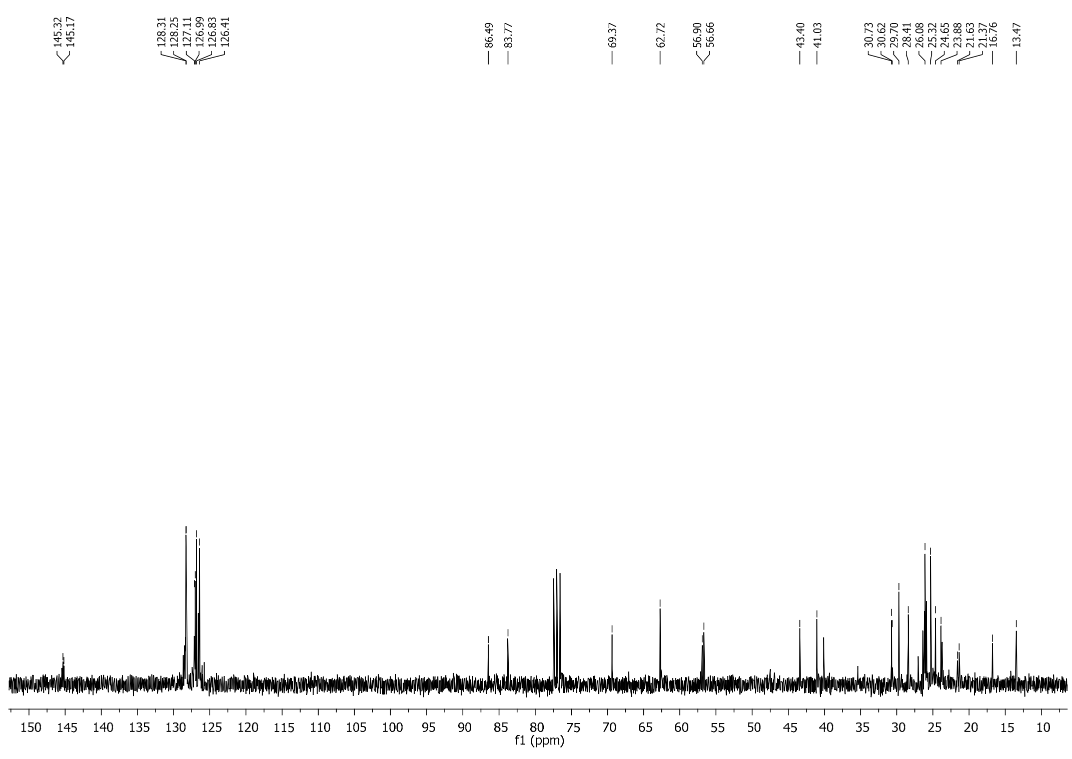

Computational Details

All optimized geometries were located using hybrid functional theory (B3LYP) [1] and the 6-31G(d, p) [2–4] basis set using the continuum solvation model [6] with chloroform (ε = 4.71) as the solvent conforming to the experimental conditions. All calculations were carried out using the Gaussian 09 program [6].

2D NMR spectra and optimized geometries to determine the absolute configuration of the new chiral centers.

To determine the absolute configuration of the new trifluoromethyl substituted chiral center ($C_β$), 2D NMR spectra and optimized geometries of new optically pure β-nitro amines compounds were obtained.

2D NMR spectra and optimized geometries

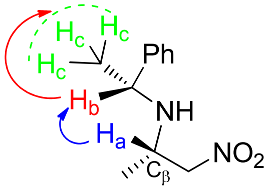

7g

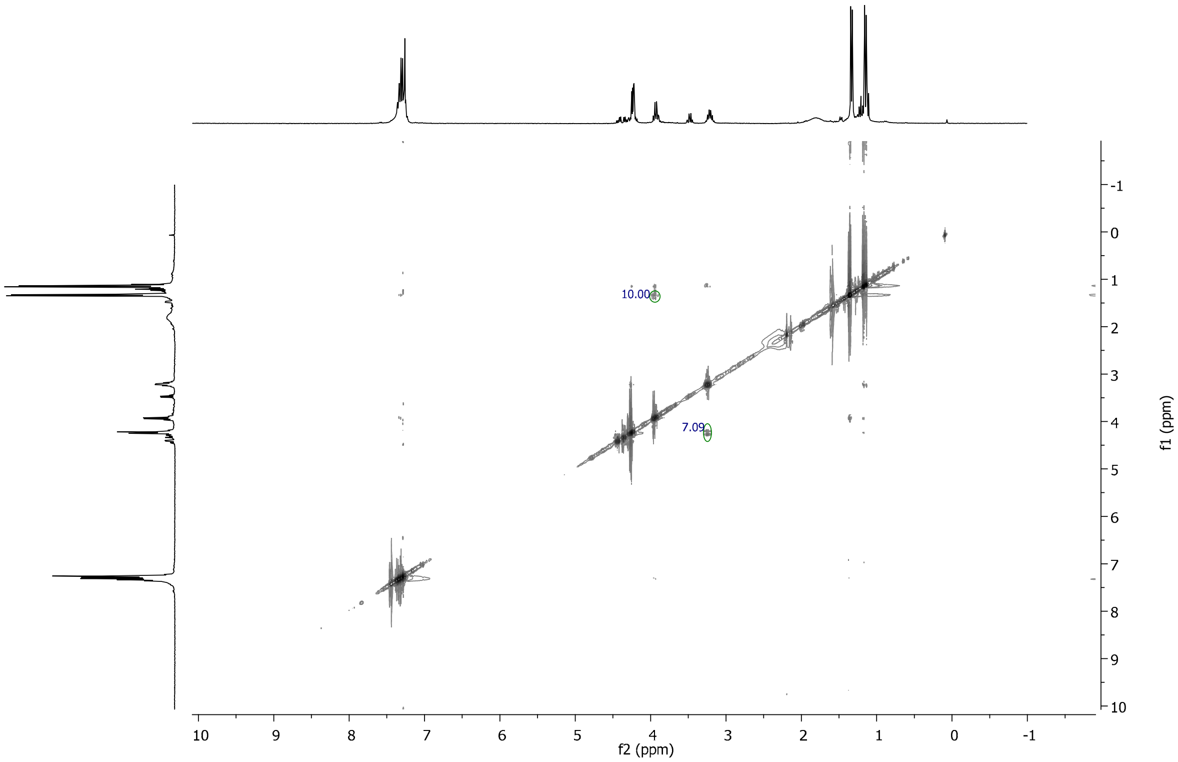

NOESY spectrum of **7g**. The cross peak between the protons H_b and H_c was used as a distance ruler and its volume was set to 10.00 a.u.; the cross peak between H_b and H_a was measured 7.09 a. u. (corresponding to an interproton distance of 2.84 Å), in order to determine the absolute configuration.

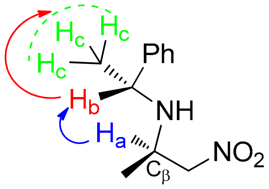

7'g

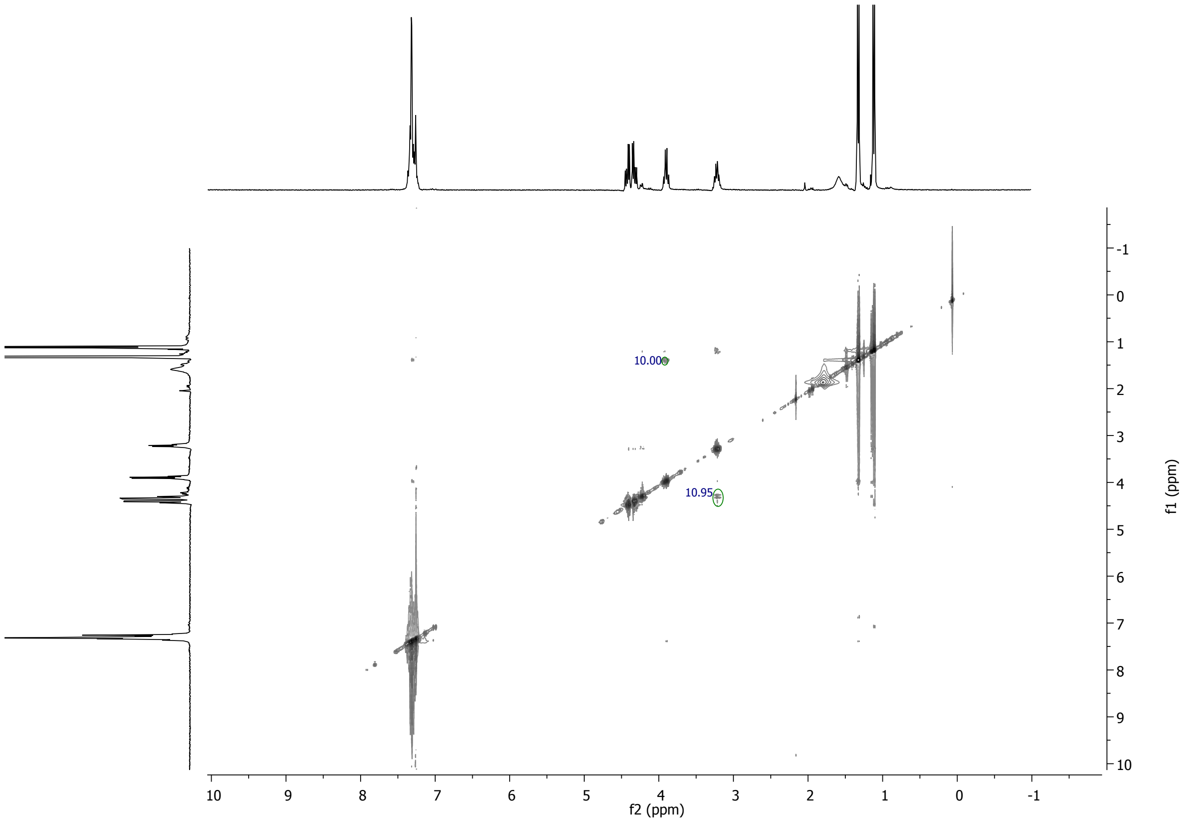

NOESY spectrum of **7'g**. The cross peak between the protons H_b and H_c was used as a distance ruler and its volume was set to 10.00 a.u.; the cross peak between H_b and H_a was measured 10.95 a. u. (corresponding to an interproton distance of 2.64 Å), in order to determine the absolute configuration.

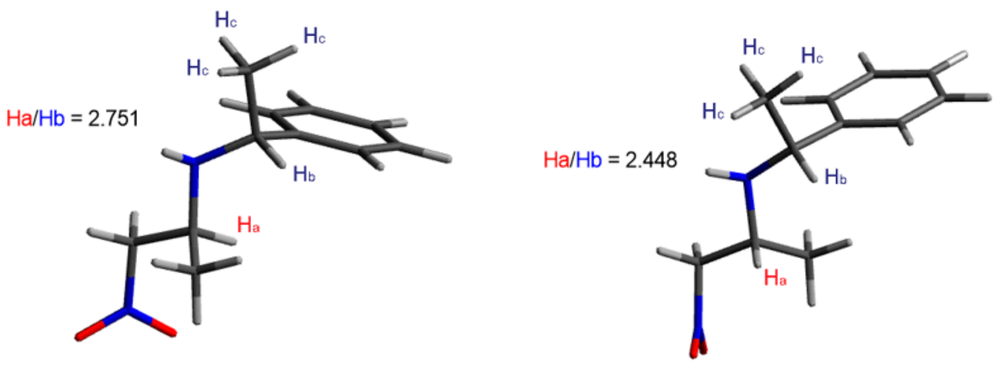

(*R*,*S*)-**7g** (*R*,*R*)-**7'g**

Figure S1. Optimized geometries of all diastereomers. By NOESY analysis coupled with computational studies absolute configurations can be assigned to C_β.

Calculated Coordinates:

(*R*,*S*)-**7g**

1	7	0	-0.376091	1.362491	-0.550683
2	6	0	0.935424	1.594949	0.076696
3	6	0	1.636075	2.740276	-0.670570
4	6	0	-1.312062	0.542742	0.228225
5	6	0	-2.309651	-0.043292	-0.784649
6	7	0	-3.279911	-0.992602	-0.134924
7	8	0	-2.808885	-1.902190	0.546287
8	8	0	-4.480336	-0.811940	-0.325692
9	6	0	1.786403	0.330086	0.055996
10	6	0	1.856332	-0.475798	-1.089082
11	6	0	2.681205	-1.600398	-1.117826
12	6	0	3.450909	-1.937478	-0.001160
13	6	0	3.386179	-1.143788	1.144874
14	6	0	2.556610	-0.020389	1.171209
15	6	0	-2.015440	1.292606	1.369033
16	1	0	-1.777874	-0.628666	-1.536795
17	1	0	-2.917743	0.722258	-1.266969
18	1	0	-0.807112	2.262839	-0.750842
19	1	0	1.249933	-0.217298	-1.951511
20	1	0	2.722872	-2.216197	-2.011980
21	1	0	4.091148	-2.814564	-0.023277
22	1	0	3.974627	-1.401344	2.020897
23	1	0	2.505386	0.591195	2.069121
24	1	0	0.834121	1.903336	1.131735
25	1	0	1.761003	2.487868	-1.727981
26	1	0	2.623186	2.930052	-0.241371
27	1	0	1.051998	3.665297	-0.601348
28	1	0	-0.744224	-0.295839	0.641749
29	1	0	-2.633230	2.109305	0.977422
30	1	0	-1.280776	1.721069	2.056406
31	1	0	-2.659433	0.627408	1.952750

(*R*,*R*)-**7′g**

1	7	0	-0.441072	1.025516	-0.549890
2	6	0	0.769770	1.400871	0.205832
3	6	0	1.280035	2.753952	-0.320231
4	6	0	-1.463508	0.290976	0.207767
5	6	0	-2.662160	0.131730	-0.748793
6	7	0	-3.843810	-0.440057	-0.021308
7	8	0	-4.200586	-1.581787	-0.303617
8	8	0	-4.370823	0.268290	0.835360
9	6	0	1.876613	0.354989	0.100031
10	6	0	2.102761	-0.344665	-1.091810
11	6	0	3.161458	-1.248011	-1.196104
12	6	0	4.012166	-1.465134	-0.109355
13	6	0	3.795000	-0.772376	1.083158
14	6	0	2.734482	0.130341	1.184076
15	1	0	-1.816572	0.867409	1.080400
16	1	0	-2.437003	-0.546477	-1.570013
17	1	0	-2.994978	1.100719	-1.127582
18	1	0	-0.866729	1.884118	-0.891168
19	1	0	1.429827	-0.184565	-1.927930
20	1	0	3.321146	-1.785949	-2.126563
21	1	0	4.833737	-2.170966	-0.189880
22	1	0	4.446588	-0.937581	1.936574
23	1	0	2.568696	0.664114	2.117458
24	1	0	0.531976	1.531370	1.275622
25	1	0	1.492855	2.689512	-1.392382
26	1	0	2.201100	3.042324	0.192065
27	1	0	0.536389	3.542429	-0.157507
28	6	0	-0.966355	-1.071978	0.695517
29	1	0	-0.603753	-1.674177	-0.142498
30	1	0	-1.770995	-1.617146	1.197624
31	1	0	-0.148412	-0.959528	1.409699

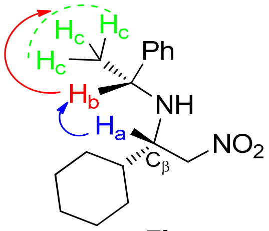

7h

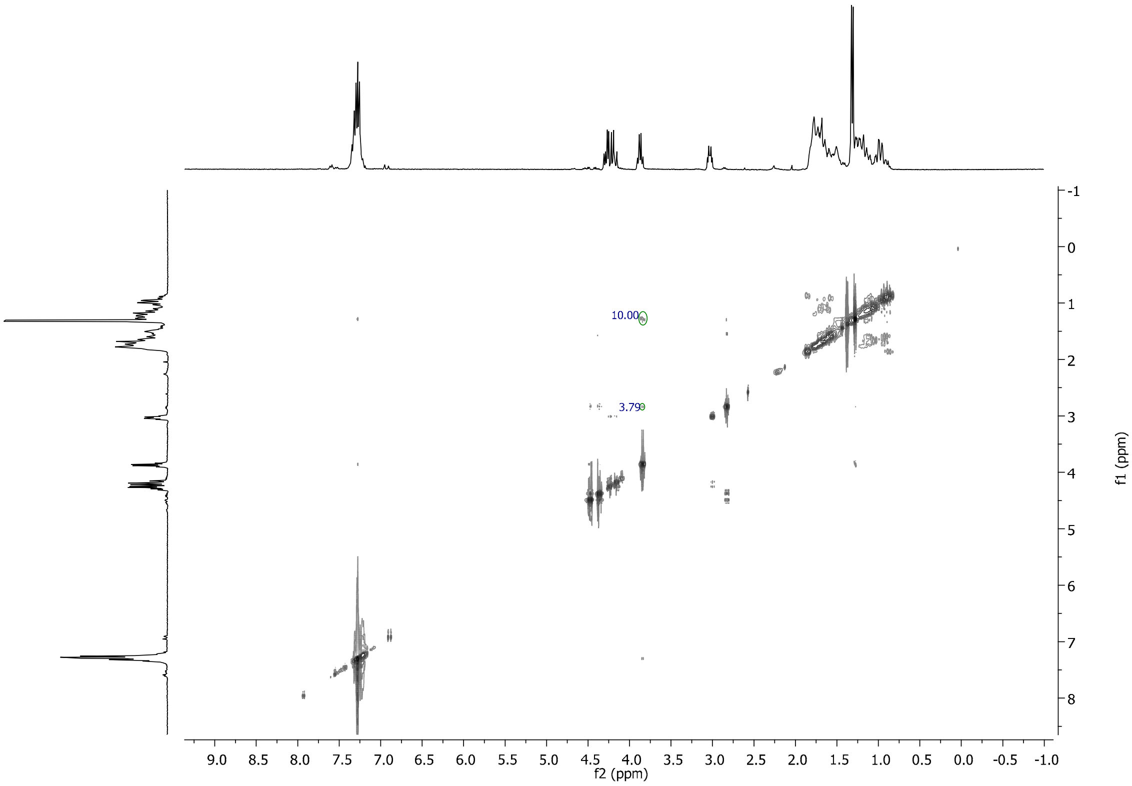

NOESY spectrum of **7h**. The cross peak between the protons H_b and H_c was used as a distance ruler and its volume was set to 10.00 a.u.; the cross peak between H_b and H_a was measured 3.79 a. u. (corresponding to an interproton distance of 3.15 Å), in order to determine the absolute configuration.

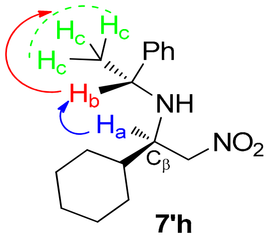

7'h

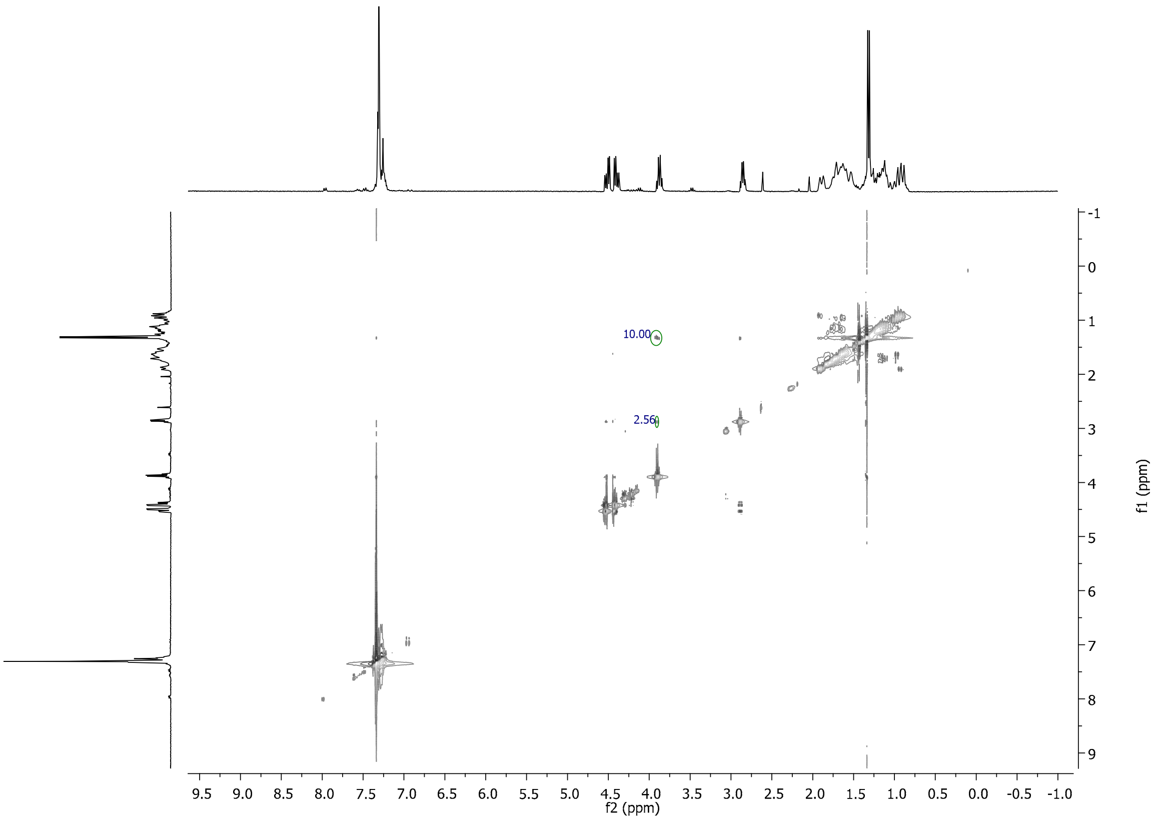

NOESY spectrum of **7'h**. The cross peak between the protons H_b and H_c was used as a distance ruler and its volume was set to 10.00 a.u.; the cross peak between H_b and H_a was measured 2.56 a. u. (corresponding to an interproton distance of 3.36 Å), in order to determine the absolute configuration.

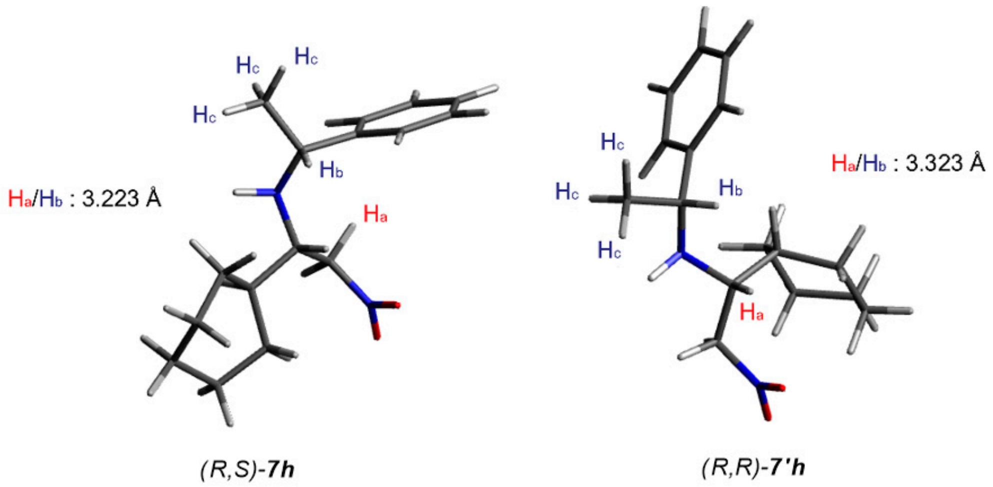

Figure S2. Optimized geometries of all diastereomers. By NOESY analysis coupled with computational studies absolute configurations can be assigned to C_β.

Calculated Coordinates:

(*R*,*S*)-**7h**

Center Number	Atomic Number	Atomic Type	Coordinates (Angstroms) X	Y	Z
1	7	0	-0.213440	-1.114666	-0.828797
2	6	0	-1.265023	-1.741176	-0.011376
3	6	0	-1.617289	-3.106151	-0.621569
4	6	0	0.551426	-0.026131	-0.192994
5	6	0	2.070150	-0.290496	-0.324833
6	6	0	2.462672	-1.585403	0.450786
7	6	0	3.927328	-1.584384	0.939347
8	6	0	4.839346	-0.886786	-0.074352
9	6	0	4.456856	0.605182	-0.214514
10	6	0	2.970073	0.878078	0.117624
11	6	0	0.087323	1.292408	-0.846489
12	7	0	0.333738	2.520914	-0.007545
13	8	0	0.132626	2.443101	1.202964
14	8	0	0.681863	3.547842	-0.588851
15	6	0	-2.506161	-0.859673	0.093861
16	6	0	-3.111115	-0.327516	-1.055009
17	6	0	-4.273500	0.437476	-0.959083
18	6	0	-4.852862	0.681935	0.289582
19	6	0	-4.258884	0.158784	1.438274
20	6	0	-3.092649	-0.604619	1.338034
21	1	0	-0.996907	1.267663	-0.970798
22	1	0	0.556706	1.486659	-1.810711
23	1	0	0.422560	-1.835540	-1.150437
24	1	0	-2.656101	-0.510315	-2.024287
25	1	0	-4.728373	0.843649	-1.858197
26	1	0	-5.756735	1.279304	0.364786
27	1	0	-4.697139	0.348889	2.413822
28	1	0	-2.631688	-1.005123	2.237704
29	1	0	-0.905691	-1.917018	1.017922
30	1	0	-1.971262	-2.990622	-1.650551
31	1	0	-2.404029	-3.593562	-0.040112
32	1	0	-0.741606	-3.765365	-0.629140
33	1	0	2.263502	-0.452945	-1.397168
34	1	0	1.795170	-1.709339	1.312503
35	1	0	2.305166	-2.461167	-0.189845

36	1	0	3.999038	-1.065794	1.903817
37	1	0	4.256276	-2.613669	1.120117
38	1	0	5.891106	-0.983267	0.216291
39	1	0	4.742512	-1.393061	-1.043907
40	1	0	5.079967	1.215709	0.448843
41	1	0	4.671515	0.944484	-1.234630
42	1	0	2.845880	1.037528	1.195917
43	1	0	2.676501	1.812310	-0.368459
44	1	0	0.299490	0.040152	0.874269

(*R*,*R*)-**7′h**

1	7	0	0.437866	1.527660	-0.534054
2	6	0	1.637761	1.642351	0.320527
3	6	0	2.348847	2.971569	0.008495
4	6	0	-0.773329	0.993161	0.121625
5	6	0	-0.594314	-0.527419	0.417355
6	6	0	-1.476451	-1.098796	1.561199
7	6	0	-2.814415	-1.737226	1.148004
8	6	0	-2.629817	-2.728220	-0.008544
9	6	0	-1.967304	-2.038832	-1.208373
10	6	0	-0.598827	-1.441791	-0.833162
11	6	0	-1.932654	1.394519	-0.808006
12	7	0	-3.289123	1.302378	-0.166942
13	8	0	-4.202243	0.826476	-0.838941
14	8	0	-3.417541	1.735654	0.975516
15	6	0	2.616567	0.483794	0.140597
16	6	0	2.864238	-0.067251	-1.123178
17	6	0	3.815234	-1.075505	-1.285892
18	6	0	4.535451	-1.548789	-0.186115
19	6	0	4.296193	-1.006423	1.077803
20	6	0	3.343751	0.002432	1.236807
21	1	0	-0.951153	1.495652	1.086768
22	1	0	-1.981706	0.808215	-1.721824
23	1	0	-1.847362	2.453956	-1.070720
24	1	0	0.227274	2.456530	-0.887463
25	1	0	2.293350	0.293733	-1.972577
26	1	0	3.993157	-1.494561	-2.272479
27	1	0	5.272745	-2.336220	-0.312773
28	1	0	4.845966	-1.370893	1.940999
29	1	0	3.159823	0.418443	2.225040
30	1	0	1.342222	1.668532	1.382664
31	1	0	2.625129	3.015594	-1.050477
32	1	0	3.262933	3.067998	0.599557
33	1	0	1.700085	3.824542	0.237233
34	1	0	-0.885263	-1.883846	2.051558
35	1	0	-1.643475	-0.327743	2.322993
36	1	0	0.084995	-2.277637	-0.635573
37	1	0	-0.165329	-0.897567	-1.679540
38	1	0	-3.545979	-0.976919	0.855281
39	1	0	-3.244592	-2.245218	2.019677
40	1	0	-3.595809	-3.156979	-0.300098
41	1	0	-1.999903	-3.566049	0.324114
42	1	0	-2.648845	-1.265026	-1.583481
43	1	0	-1.831918	-2.750981	-2.031472
44	1	0	0.425147	-0.566294	0.812118

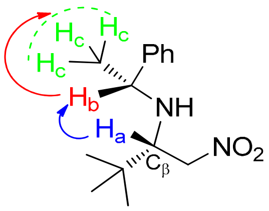

7i

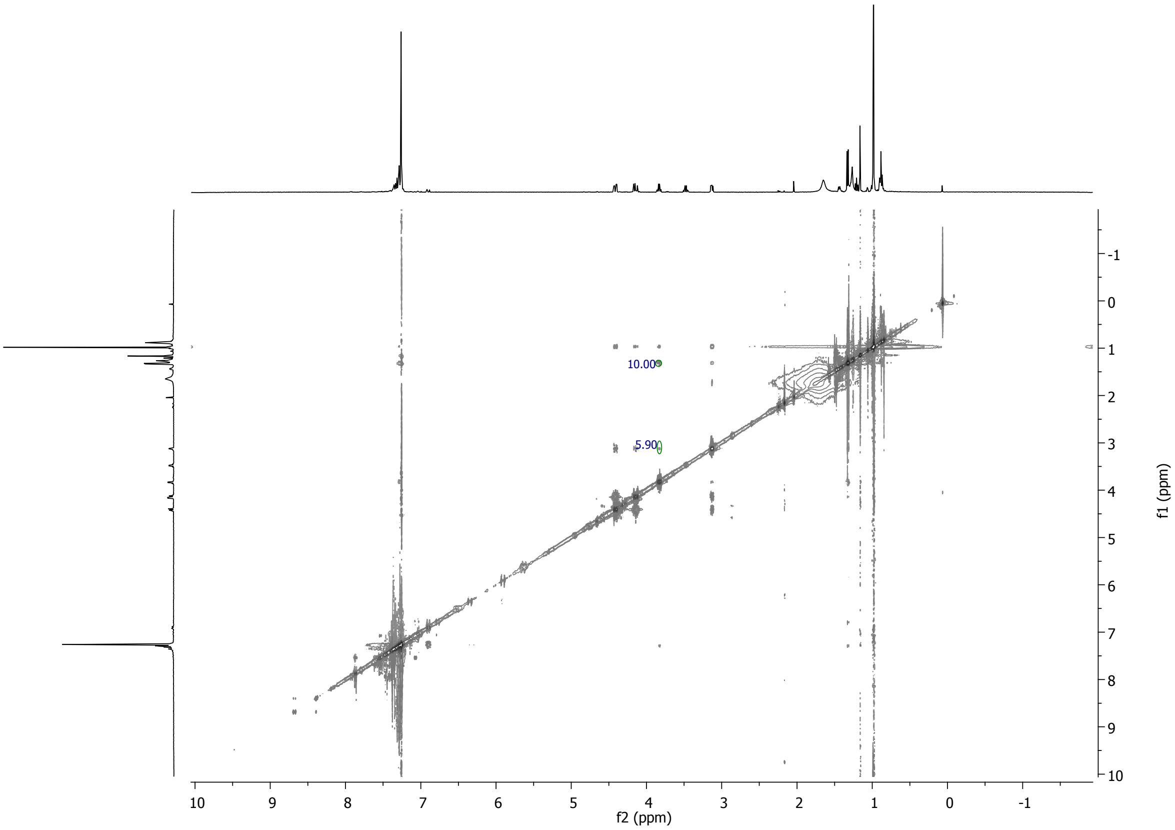

NOESY spectrum of **7i**. The cross peak between the protons H_b and H_c was used as a distance ruler and its volume was set to 10.00 a.u.; the cross peak between H_b and H_a was measured 5.90 a. u. (corresponding to an interproton distance of 2.92 Å), in order to determine the absolute configuration.

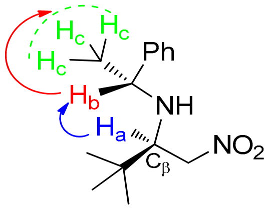

7'i

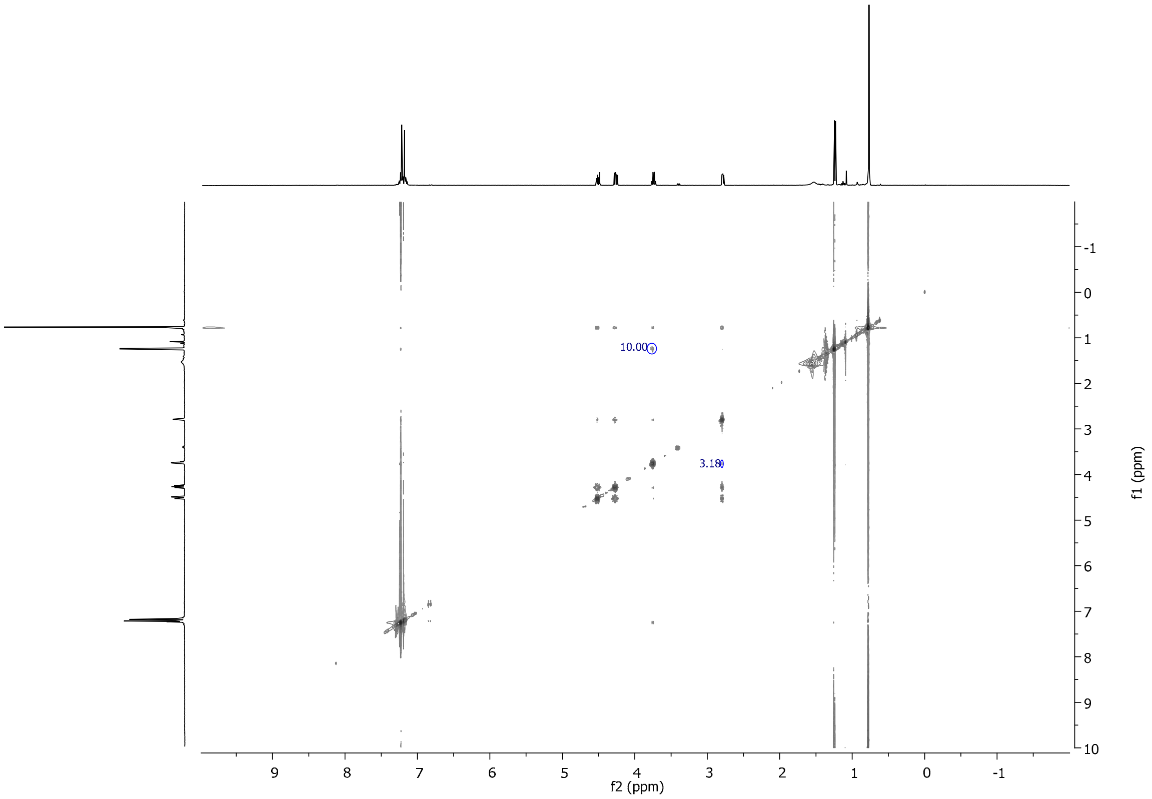

NOESY spectrum of ***7'i***. The cross peak between the protons H_b and H_c was used as a distance ruler and its volume was set to 10.00 a.u.; the cross peak between H_b and H_a was measured 3.18 a. u. (corresponding to an interproton distance of 3.24 Å), in order to determine the absolute configuration.

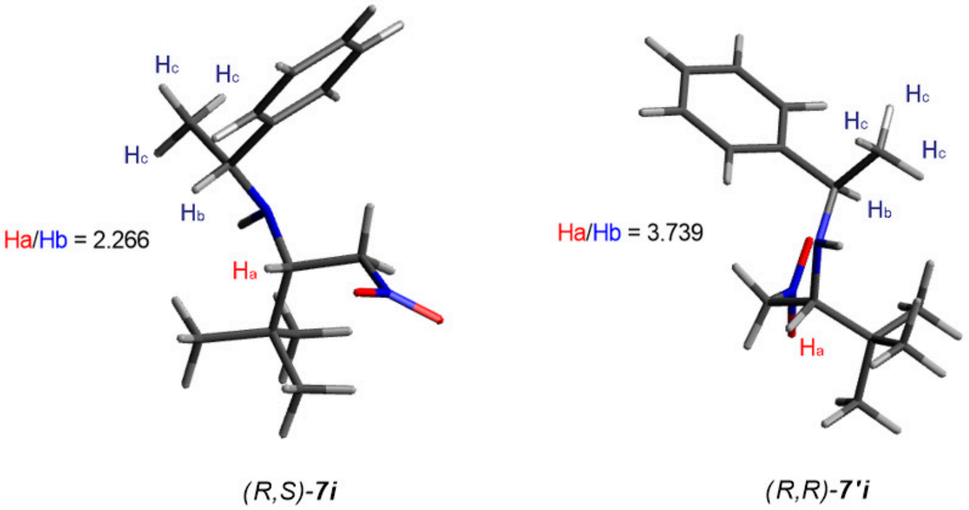

Figure S3. Optimized geometries of all diastereomers. By NOESY analysis coupled with computational studies absolute configurations can be assigned to C_{β}.

Calculated Coordinates:

(*R*,*S*)-**7i**

Center Number	Atomic Number	Atomic Type	Coordinates (Angstroms) X	Y	Z
1	6	0	-2.663933	-0.206895	1.283850
2	6	0	-2.017982	-0.687757	0.139884
3	6	0	-2.470957	-0.258861	-1.117004
4	6	0	-3.543302	0.626715	-1.224020
5	6	0	-4.183349	1.097541	-0.073719
6	6	0	-3.740215	0.678494	1.180918
7	6	0	-0.882674	-1.700545	0.258570
8	6	0	-1.349856	-3.091520	-0.197485
9	7	0	0.291684	-1.307351	-0.537496
10	6	0	1.137533	-0.248412	0.039168
11	6	0	0.863312	1.037316	-0.776126
12	7	0	1.156912	2.317648	-0.036782
13	8	0	0.777148	2.407997	1.129344
14	6	0	2.645927	-0.664198	0.111615
15	6	0	3.199954	-1.079378	-1.267958
16	6	0	2.785080	-1.844758	1.097586
17	6	0	3.498847	0.503185	0.651014
18	8	0	1.722451	3.218800	-0.654413
19	1	0	0.813912	-0.034730	1.067250
20	1	0	-0.205595	1.093321	-0.987716
21	1	0	1.419385	1.095183	-1.709819
22	1	0	0.847060	-2.133218	-0.725627
23	1	0	-1.967910	-0.617590	-2.010390
24	1	0	-3.880494	0.950388	-2.204751
25	1	0	-5.017133	1.788492	-0.156619
26	1	0	-4.226171	1.043959	2.081007
27	1	0	-2.320070	-0.525741	2.264897
28	1	0	-0.620623	-1.769612	1.328948
29	1	0	-1.608801	-3.084136	-1.260699
30	1	0	-2.232043	-3.401901	0.368137
31	1	0	-0.563737	-3.838594	-0.037985
32	1	0	2.424342	-1.567951	2.094096
33	1	0	3.836241	-2.135590	1.191146
34	1	0	2.230208	-2.731582	0.775232

35	1	0	3.125878	0.863302	1.615070
36	1	0	3.531716	1.348229	-0.042609
37	1	0	4.531131	0.168948	0.796074
38	1	0	2.666429	-1.931596	-1.701103
39	1	0	4.250247	-1.372638	-1.170355
40	1	0	3.158748	-0.258972	-1.991448

(*R*,*R*)-**7'i**

Center Number	Atomic Number	Atomic Type	Coordinates (Angstroms) X	Y	Z
1	7	0	0.295280	-1.226288	-0.608629
2	6	0	1.401163	-0.309686	-0.891840
3	6	0	2.757402	-0.528053	-0.111996
4	6	0	2.658193	-0.324815	1.413401
5	6	0	3.832839	0.415237	-0.693425
6	6	0	3.214477	-1.980631	-0.370895
7	6	0	0.920725	1.157784	-1.056608
8	7	0	0.944093	2.054060	0.160435
9	1	0	-0.118135	1.153712	-1.388980
10	1	0	1.544710	1.681727	-1.775827
11	8	0	1.769540	2.967644	0.172434
12	8	0	0.135426	1.850605	1.062232
13	6	0	-0.612569	-1.106376	0.540403
14	1	0	0.600009	-2.181462	-0.739508
15	6	0	-1.964217	-0.497058	0.148097
16	6	0	-2.484734	-0.634058	-1.143894
17	1	0	-1.880653	-1.131016	-1.896342
18	6	0	-3.747635	-0.128729	-1.463510
19	1	0	-4.134792	-0.243681	-2.472475
20	6	0	-4.510272	0.522920	-0.492873
21	1	0	-5.490599	0.919560	-0.741003
22	6	0	-3.998738	0.667994	0.799483
23	1	0	-4.579425	1.182326	1.560270
24	6	0	-2.736861	0.162987	1.113577
25	1	0	-2.340810	0.290634	2.118233
26	1	0	-0.183875	-0.462436	1.313509
27	6	0	-0.836418	-2.499367	1.156411
28	1	0	-1.227348	-3.191321	0.401437
29	1	0	-1.562964	-2.452361	1.971551
30	1	0	0.100066	-2.907848	1.551920
31	1	0	2.412285	0.700930	1.696194
32	1	0	3.623698	-0.561458	1.873881
33	1	0	1.914181	-0.990635	1.859529
34	1	0	3.621578	1.465743	-0.479730
35	1	0	3.918174	0.296853	-1.780375
36	1	0	4.810752	0.180019	-0.260650
37	1	0	2.529933	-2.712762	0.069261
38	1	0	4.198902	-2.149838	0.076880
39	1	0	3.297130	-2.191462	-1.443644
40	1	0	1.671716	-0.544532	-1.932085

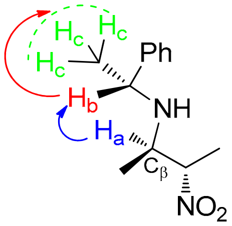

(R,R,S)-anti-**9'g**

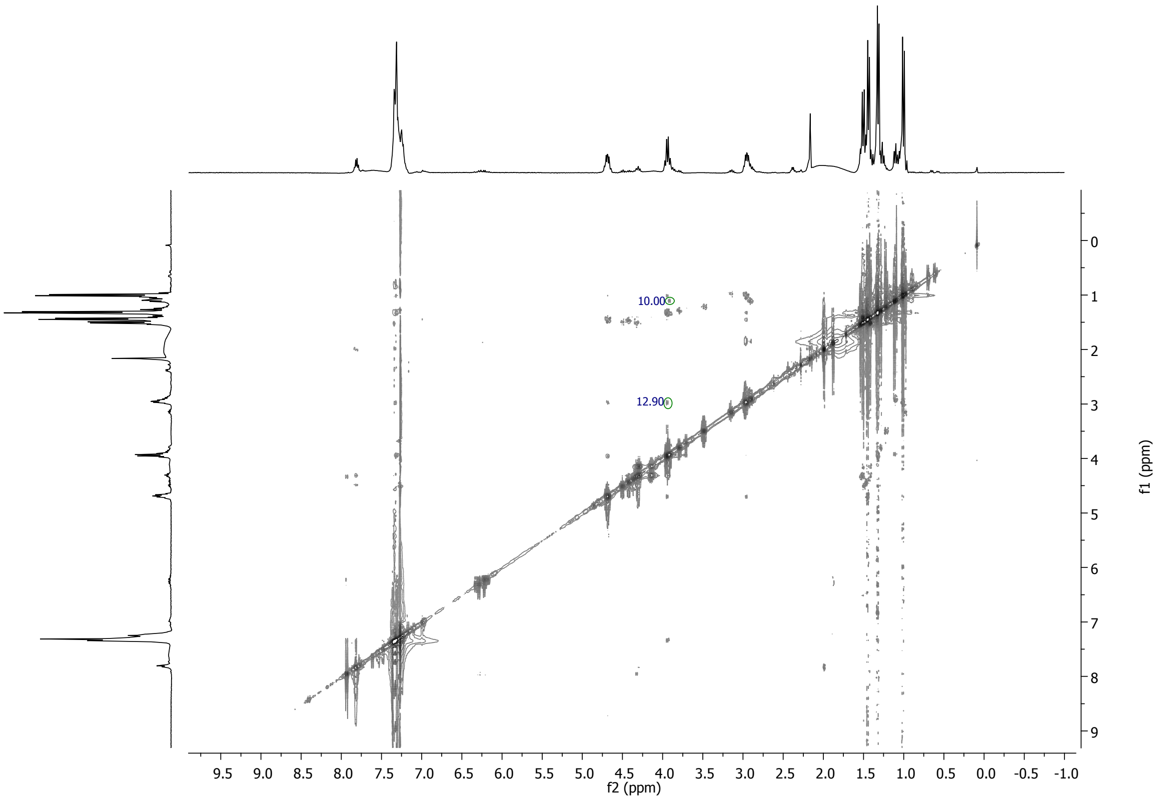

NOESY spectrum of anti-**9′g**. The cross peak between the protons H_b and H_c was used as a distance ruler and its volume was set to 10.00 a.u.; the cross peak between H_b and H_a was measured 12.90 a. u. (corresponding to an interproton distance of 2.56 Å), in order to determine the absolute configuration.

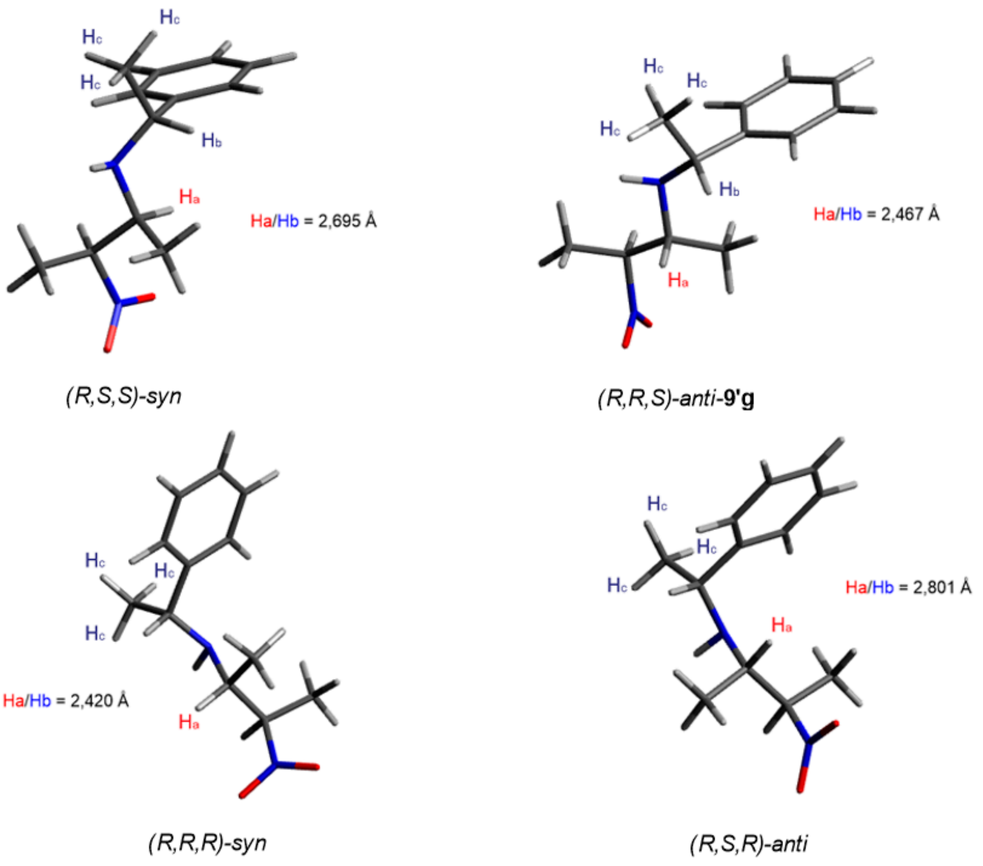

Figure S4. Optimized geometries of all diastereomers. By NOESY analysis coupled with computational studies absolute configurations can be assigned to C_{β}.

Calculated Coordinates:

(*R*,*S*,*S*)-*syn*

1	7	0	-0.260449	1.380576	-0.333699
2	6	0	1.068448	1.592715	0.265234
3	6	0	1.708780	2.822528	-0.396926
4	6	0	-1.141266	0.464926	0.400941
5	6	0	-2.105035	-0.180591	-0.637714
6	7	0	-2.879479	-1.278634	0.075924
7	8	0	-2.238716	-2.272133	0.417110
8	8	0	-4.075881	-1.113254	0.304484
9	6	0	1.951571	0.361333	0.097822
10	6	0	1.999073	-0.335219	-1.117919
11	6	0	2.852236	-1.427096	-1.278820
12	6	0	3.672987	-1.840366	-0.225937
13	6	0	3.630878	-1.155892	0.989500
14	6	0	2.773239	-0.064934	1.147967
15	6	0	-1.864824	1.114077	1.590329
16	1	0	-1.484746	-0.725464	-1.351380
17	6	0	-3.047326	0.774464	-1.351102
18	1	0	-0.719407	2.283472	-0.429764
19	1	0	1.353189	-0.018011	-1.930738
20	1	0	2.875785	-1.958292	-2.226275
21	1	0	4.334868	-2.692379	-0.350911
22	1	0	4.258973	-1.473631	1.816879
23	1	0	2.740345	0.461117	2.099303

24	1	0	0.997911	1.799517	1.347365
25	1	0	1.801529	2.670950	-1.476637
26	1	0	2.705361	3.003500	0.013764
27	1	0	1.101899	3.718779	-0.223346
28	1	0	-0.516424	-0.353529	0.770946
29	1	0	-2.498952	1.947054	1.269188
30	1	0	-1.132289	1.506529	2.301292
31	1	0	-2.494172	0.399152	2.128932
32	1	0	-3.678624	0.238954	-2.063681
33	1	0	-2.445497	1.496984	-1.907855
34	1	0	-3.696042	1.309142	-0.655660

(*R*,*R*,*S*)- *anti*-**9**|**g**

1	7	0	0.278066	1.018887	0.311285
2	6	0	-0.949371	1.307884	-0.457537
3	6	0	-1.406636	2.740901	-0.132043
4	6	0	1.266201	0.162219	-0.359430
5	6	0	2.496551	0.054935	0.580872
6	7	0	3.535761	-0.806253	-0.111399
7	8	0	3.794350	-1.903215	0.378433
8	8	0	4.054761	-0.357846	-1.132933
9	6	0	-2.079969	0.326604	-0.158083
10	6	0	-2.272346	-0.189993	1.129139
11	6	0	-3.352414	-1.030234	1.404143
12	6	0	-4.257777	-1.366711	0.394848
13	6	0	-4.074164	-0.857111	-0.891958
14	6	0	-2.992510	-0.016914	-1.163471
15	1	0	1.621324	0.614609	-1.302561
16	1	0	2.228124	-0.511019	1.473615
17	6	0	3.156100	1.384378	0.946956
18	1	0	0.722179	1.910961	0.504775
19	1	0	-1.556795	0.061792	1.905095
20	1	0	-3.485831	-1.425750	2.407335
21	1	0	-5.095802	-2.023905	0.608185
22	1	0	-4.768556	-1.117421	-1.685859
23	1	0	-2.852769	0.373585	-2.169233
24	1	0	-0.745949	1.265746	-1.541242
25	1	0	-1.582308	2.849040	0.943397
26	1	0	-2.338156	2.975462	-0.652981
27	1	0	-0.649967	3.472161	-0.438757
28	6	0	0.698673	-1.225318	-0.675267
29	1	0	0.359022	-1.724097	0.237086
30	1	0	1.452402	-1.852377	-1.158943
31	1	0	-0.151277	-1.154136	-1.356010
32	1	0	4.090391	1.213930	1.488534
33	1	0	2.498333	1.950357	1.610267
34	1	0	3.371925	1.977278	0.055092

(*R*,*R*,*R*)-*syn*

1	7	0	0.281452	0.972226	0.572877
2	6	0	-0.946517	1.441552	-0.098843
3	6	0	-1.437094	2.715998	0.610125
4	6	0	1.277481	0.329507	-0.296619
5	6	0	2.552418	0.116369	0.572730
6	7	0	3.690465	-0.238121	-0.372720
7	8	0	4.070971	-1.404698	-0.433924
8	8	0	4.147679	0.678976	-1.053518
9	6	0	-2.055237	0.392420	-0.101373
10	6	0	-2.280495	-0.427822	1.011335

11	6	0	-3.341854	-1.333773	1.024145
12	6	0	-4.196587	-1.433225	-0.076657
13	6	0	-3.980328	-0.620274	-1.190717
14	6	0	-2.916994	0.285049	-1.199824
15	1	0	1.579996	1.001364	-1.118683
16	6	0	2.427437	-0.901722	1.692823
17	1	0	2.859535	1.091059	0.961958
18	1	0	0.723053	1.783687	0.999345
19	1	0	-1.606079	-0.358005	1.858637
20	1	0	-3.500861	-1.965408	1.893854
21	1	0	-5.020331	-2.141058	-0.067544
22	1	0	-4.634944	-0.693196	-2.054614
23	1	0	-2.752292	0.913729	-2.072234
24	1	0	-0.734258	1.708002	-1.148772
25	1	0	-1.624455	2.513121	1.669656
26	1	0	-2.368456	3.071151	0.162302
27	1	0	-0.693002	3.517019	0.533262
28	6	0	0.760012	-0.971377	-0.916098
29	1	0	0.373588	-1.650161	-0.152451
30	1	0	1.557076	-1.478084	-1.467671
31	1	0	-0.050485	-0.765684	-1.618182
32	1	0	3.337439	-0.930101	2.296879
33	1	0	2.237306	-1.904227	1.308886
34	1	0	1.591082	-0.602386	2.327649

(*R*,*S*,*R*)-*anti*

1	7	0	0.251217	1.350612	0.557980
2	6	0	-1.068681	1.635627	-0.032832
3	6	0	-1.751441	2.725672	0.807369
4	6	0	1.169204	0.573896	-0.283485
5	6	0	2.246927	-0.034733	0.651616
6	7	0	3.240509	-0.803752	-0.199241
7	8	0	2.825184	-1.792116	-0.802876
8	8	0	4.398837	-0.394503	-0.246876
9	6	0	-1.926624	0.377514	-0.101816
10	6	0	-2.084634	-0.452016	1.017729
11	6	0	-2.909750	-1.574910	0.961466
12	6	0	-3.592256	-1.888246	-0.217553
13	6	0	-3.440619	-1.071385	-1.338268
14	6	0	-2.611240	0.051505	-1.278240
15	6	0	1.794246	1.383227	-1.432259
16	6	0	1.695746	-0.972772	1.720004
17	1	0	2.848603	0.761205	1.095973
18	1	0	0.694270	2.234534	0.802793
19	1	0	-1.549517	-0.213968	1.931495
20	1	0	-3.020523	-2.207675	1.837656
21	1	0	-4.232970	-2.764122	-0.261817
22	1	0	-3.961373	-1.309147	-2.261455
23	1	0	-2.493641	0.681727	-2.156669
24	1	0	-0.979470	2.023561	-1.062049
25	1	0	-1.865953	2.397427	1.844820
26	1	0	-2.741943	2.953478	0.405207
27	1	0	-1.161576	3.649561	0.800230
28	1	0	0.598078	-0.262185	-0.700488
29	1	0	2.435464	2.183053	-1.044064
30	1	0	1.012000	1.842105	-2.042712
31	1	0	2.396522	0.756788	-2.096768
32	1	0	2.502297	-1.380427	2.335635
33	1	0	1.144399	-1.796948	1.262040
34	1	0	1.015822	-0.404292	2.354639

References

1. Becke, A.D. A new mixing of Hartree-Fock and local density-functional theories. *J. Chem. Phys*. **1993**, *98*, 1372–1377.
2. Ditchfield, R.; Hehre, W.J.; Pople, J.A. Self-Consistent Molecular-Orbital Methods. IX. An Extended Gaussian-Type Basis for Molecular-Orbital Studies of Organic Molecules. *J. Chem. Phys.* **1971**, *54*, 724–728.
3. Hehre, W. J.; Ditchfield, R.; Pople, J.A. Self-Consistent Molecular Orbital Methods. XII. Further Extensions of Gaussian-Type Basis Sets for Use in Molecular Orbital Studies of Organic Molecules. *J. Chem. Phys.* **1972**, *56*, 2257–2261.
4. Hariharan, P.C.; Pople, J.A. Influence of polarization functions on MO hydrogenation energies. *Theor. Chim. Acta* **1973**, *28*, 213–223.
5. Barone, V.; Cossi, M.; Tomasi, J. Geometry optimization of molecular structures in solution by the polarizable continuum model. *J. Comput. Chem.* **1998**, *19*, 404–417.
6. Frisch, M. J.; Trucks, G. W.; Schlegel, H. B.; Scuseria, G. E.; Robb, M. A.; Cheeseman, J. R.; Scalmani, G.; Barone, V.; Mennucci, B.; Petersson, G. A.; *et al. Gaussian 09, Revision D.01*; Gaussian, Inc.: Wallingford, CT, USA, 2013.
